# Supplementary material for: DNA methylation signature of chronic low-grade inflammation and its role in cardio-respiratory diseases
Source: Nat Commun. 2022 May 3;13:2408. doi: 10.1038/s41467-022-29792-6 (PMC9065016; doi:10.1038/s41467-022-29792-6)
Supplement: Supplementary file 1 — Supplementary Information [file 41467_2022_29792_MOESM1_ESM.pdf]

Description of participating cohorts:

Airwave - The Airwave Health Monitoring Study

Airwave - The Airwave Health Monitoring Study is an occupational cohort of employees of 28 police forces from across Great Britain. Full details of the cohort and methods are available in Elliott et al[1]. The study started recruitment in 2006 and now contains 53,280 participants. At the baseline health screening, participants underwent health examination, self-completed a computer questionnaire and blood samples were collected in EDTA tubes for DNA extraction.

Ethics

The study received ethical approval from the National Health Service Multi-Site Research Ethics Committee (MREC/13/NW/0588).

DNA Methylation

For the microarray, bisulphite conversion of 500 ng of each DNA sample was performed using the EZ DNA Methylation-Lightning™ Kit according to the manufacturer's protocol (Zymo Research, Orange, CA). Then, bisulfite-converted DNA was used for hybridization on the Infinium HumanMethylation EPIC BeadChip, following the Illumina Infinium HD Methylation protocol. Briefly, a whole genome amplification step was followed by enzymatic end-point fragmentation and hybridization to HumanMethylation EPIC BeadChips at 48°C for 17 h, followed by single nucleotide extension. The incorporated nucleotides were labelled with biotin (ddCTP and ddGTP) and 2,4-dinitrophenol (DNP) (ddATP and ddTTP). After the extension step and staining, the BeadChip was washed and scanned using the Illumina HiScan SQ scanner. The intensities of the images were extracted using the GenomeStudio (v.2011.1) Methylation module (1.9.0) software, which normalizes within-sample data using different internal controls that are present on the HumanMethylation EPIC BeadChip and internal background probes. The methylation score for each CpG was represented as a β-value according to the fluorescent intensity ratio representing any value between 0 (unmethylated) and 1 (completely methylated).

DNA methylation (DNAm) data were pre-processed and normalized using in-house software written for the R statistical computing environment, including background and color bias correction, quantile normalization, and Beta MIxture Quantile dilation (BMIQ) procedure to remove type I/type II probes bias, as described elsewhere[2]. DNAm levels were expressed as

the ratio of the intensities of methylated cytosines over the total intensities (β values). Cross-reactive and polymorphic probes - with minor allele frequency greater than 0.01 in Europeans[3] - were excluded. Methylation measures were set to missing if the detection p-value was greater than 0.01. Samples with the bisulfite conversion control fluorescence intensity lower than 10,000 for both type I and type II probes and those with total call rate lower than 95% were excluded. Finally, samples were excluded if the predicted sex (based on chromosome X methylation) did not match that self-reported.

Genotyping, imputation and quality control

Genotyping was performed on the Illumina Infinium HumanCoreExome-12v1-1 BeadChip and quality control filters including call rate (>=97%), heterozygosity rate (<=3SD from the mean) were applied on the samples. Duplicated and second-degree relatives were further excluded and 14,062 samples of European ancestry based on principle component analysis remained. Markers were removed for high missing rate (>2%), deviation from Hardy-Weinberg equilibrium (P<1E-5) or minor allele frequency below 1%, resulting in 254,027 high-quality and common markers. Imputation was performed using the Haplotype Reference Consortium (HRC) panel (version r1.1 2016).

Acknowledgements

OR was supported by an UK Research and Innovation Future Leaders Fellowship (MR/S03532X/1). This study was partly supported by the European Commission grant to the LIFEPATH project (Horizon 2020 grant number 633666). The Airwave Health Monitoring Study was funded by the Home Office (2003-2018, grant number 780- TETRA) and is currently funded by the Medical Research Council/Economic & Social Research Council (grant number MR/R023484/1) with additional support from the National Institute for Health Research (NIHR) Imperial College Biomedical Research Centre. The Airwave Study uses the computing resources of the UK MEDical BIOinformatics partnership (UK MED-BIO supported by the Medical Research Council (MR/L01632X/1). We thank all Airwave participants for their contributions. PE is Director of the MRC Centre for Environment and Health and acknowledges support from the Medical Research Council (Mr/S019669/1). PE also acknowledges support from the Imperial College BHF Centre for Research Excellence (RE/18/4/34215).

ALSPAC (ARIES)

Pregnant women resident in Avon, UK with expected dates of delivery 1st April 1991 to 31st December 1992 were invited to take part in the study[4-6]. The initial number of pregnancies enrolled is 14,541 (for these at least one questionnaire has been returned or a "Children in Focus" clinic had been attended by 19/07/99). Of these initial pregnancies, there was a total of 14,676 foetuses, resulting in 14,062 live births and 13,988 children who were alive at 1 year of age.

When the oldest children were approximately 7 years of age, an attempt was made to bolster the initial sample with eligible cases who had failed to join the study originally. As a result, when considering variables collected from the age of seven onwards (and potentially abstracted from obstetric notes) there are data available for more than the 14,541 pregnancies mentioned above. The number of new pregnancies not in the initial sample (known as Phase I enrolment) that are currently represented on the built files and reflecting enrolment status at the age of 24 is 913 (456, 262 and 195 recruited during Phases II, III and IV respectively), resulting in an additional 913 children being enrolled. The phases of enrolment are described in more detail in the cohort profile paper and its update (see footnote 4 below). The total sample size for analyses using any data collected after the age of seven is therefore 15,454 pregnancies, resulting in 15,589 foetuses. Of these 14,901 were alive at 1 year of age.

Please note that the study website contains details of all the data that is available through a fully searchable data dictionary and variable search tool" and reference the following webpage: http://www.bristol.ac.uk/alspac/researchers/our-data/

Ethical approval for the study was obtained from the ALSPAC Ethics and Law Committee and the Local Research Ethics Committees. Consent for biological samples has been collected in accordance with the Human Tissue Act (2004).

Funding:

The UK Medical Research Council and Wellcome (Grant ref: 217065/Z/19/Z) and the University of Bristol provide core support for ALSPAC. This publication is the work of the authors and MW will serve as guarantors for the contents of this paper.

This research was funded in whole, or in part, by the Wellcome Trust [Grant number]. For the purpose of Open Access, the author has applied a CC BY public copyright licence to any Author Accepted Manuscript version arising from this submission. A comprehensive list of grants funding is available on the ALSPAC website (http://www.bristol.ac.uk/alspac/external/documents/grant-acknowledgements.pdf); This research was specifically funded by WT092830/Z/10/Z; BBI025751/1 and BB/I025263/1; MC_UU_00011/5; and G1001357 and supported by the European Union’s Horizon 2020 research and innovation programme (Grant no. 848158).

Acknowledgement:

We are extremely grateful to all the families who took part in this study, the midwives for their help in recruiting them, and the whole ALSPAC team, which includes interviewers, computer and laboratory technicians, clerical workers, research scientists, volunteers, managers, receptionists and nurses.”

The Atherosclerosis Risk in Communities study (ARIC)

The ARIC Study is an ongoing prospective cohort study in four US communities[7]. A total of 15,792 participants aged 45–64 years were recruited from Forsyth County, North Carolina; Jackson, Mississippi (African Americans only); suburban Minneapolis, Minnesota; and Washington County, Maryland between 1987 and 1989 (Visit 1). Regular follow-up examinations were conducted and are still ongoing. Measures of DNA methylation in peripheral blood leukocyte samples were available for 2,879 African Americans study participants from Visit 2 (1990–92) and Visit 3 (1993–95).

Quantification of DNA methylation was described previously[8]. In brief, for the quantification of DNA methylation in the ARIC study, genomic DNA was extracted from peripheral blood leukocyte samples. Levels of DNA methylation were quantified using the Illumina Infinium HumanMethylation450K Beadchip array (HM450K). Illumina GenomeStudio Methylation module 1.9.0 was used to extract the intensity value of each site and perform background correction. The Beta Mixture Quantile Dilation (BMIQ) method was used to adjust the beta values of type 2 design probes on the array to the statistical distribution characteristic of type 1 probes[9]. We excluded probe sites with detection P-value >0.01, beadcount <3 in ≥5% of the sample and missing in ≥1% of the sample, resulting in a total of 480,407 sites for analysis. We further excluded samples (n = 83) having ≥1% of the probe sites with detection

P-value >0.01 or missing, SNP mismatch between HM450K array and microarray data (Affymetric 6.0, Exome Chip, IBC chip, Metabochip), or outliers in multi-dimensional scaling analysis. After quality control and intersection with covariates, there were a total of 2,182 samples and 480,407 CpG sites available for analysis.

Acknowledgements and funding sources

The Atherosclerosis Risk in Communities study has been funded in whole or in part with Federal funds from the National Heart, Lung, and Blood Institute, National Institutes of Health, Department of Health and Human Services (contract numbers HHSN268201700001I, HHSN268201700002I, HHSN268201700003I, HHSN268201700004I and HHSN268201700005I), R01HL087641, R01HL059367 and R01HL086694; National Human Genome Research Institute contract U01HG004402; and National Institutes of Health contract HHSN268200625226C. Funding was also supported by 5RC2HL102419, R01NS087541 and R01HL131136. The authors thank the staff and participants of the ARIC study for their important contributions. Infrastructure was partly supported by Grant Number UL1RR025005, a component of the National Institutes of Health and NIH Roadmap for Medical Research. The work of Anna Köttgen was funded by the Deutsche Forschungsgemeinschaft (DFG, German Research Foundation) Heisenberg Professorship (KO 3598/5-1), and Project-ID 192904750 – SFB 992. The work of Pascal Schlosser was funded by DFG Project-ID 192904750 – SFB 992.

BIOS cohorts:

BIOS: Rotterdam Study (RS)

RS is a large prospective, population-based cohort study aimed at assessing the occurrence of and risk factors for chronic (cardiovascular, endocrine, hepatic, neurological, ophthalmic, psychiatric, dermatological, oncological, and respiratory) diseases in the elderly[10,11]. The study comprises 14,926 subjects in total, living in the well-defined Ommoord district in the city of Rotterdam in the Netherlands. In 1989, the first cohort, Rotterdam Study-I (RS-I) comprised of 7,983 subjects with age 55 years or above. In 2000, the second cohort, Rotterdam Study-II (RS-II) was included with 3,011 subjects who had reached an age of 55 or over in 2000. In 2006, the third cohort, Rotterdam Study-III (RS-III) was further included with 3,932 subjects with age 45 years and above.

BIOS: Leiden Longevity Study (LLS)

The aim of LLS[12] is to identify genetic factors influencing longevity and examine their interaction with the environment to develop interventions by which to increase health at older ages. To this end, long-lived siblings of European descent were recruited together with their offspring and their offspring's partners, on the condition that at least two long-lived siblings were alive at the time of ascertainment. For men, the age criterion was 89 years or older; for women, the age criterion was 91 years or older. These criteria led to the ascertainment of 944 long-lived siblings from 421 families, together with 1,671 of their offspring and 744 partners.

BIOS: LifeLines-DEEP (LLD)

The LLD cohort[13] is a sub-cohort of the LifeLines cohort[14] with additional molecular data on 1,500 participants. LifeLines is a multi-disciplinary prospective population-based cohort study examining the health and health-related behaviours of 167,729 individuals living in the northern parts of The Netherlands using a unique three-generation design. It employs a broad range of investigative procedures assessing the biomedical, socio-demographic, behavioural, physical and psychological factors contributing to health and disease in the general population, with a special focus on multi-morbidity and complex genetics.

BIOS: Netherlands Twin Register (NTR)

The NTR was founded on February 1st 1987 at the Vrije Universiteit in Amsterdam. A large number of families with young twins are registered and followed from birth in their development. An important research of the NTR focuses on the health and lifestyles of adolescents and adults[15]. Approximately 25,000 twins and multiples over 18 years and 62,000 twins and multiples between 0 and 18 years are registered with the NTR. Overall, over 175,000 subjects (multiples, parents, siblings, spouses etc.) are registered. The aim of the NTR is to examine the contribution of hereditary predisposition to personality, growth, development, disease and risk factors for disease. Multiples are not different from singles, but with the help of twins, we can determine to what extent differences between individuals are contributed by heredity and environmental factors.

BIOS: Cohort on Diabetes and Atherosclerosis Maastricht (CODAM)

The CODAM cohort consists of over 500 individuals (301 with normal glucose tolerance; 127 with impaired glucose metabolism, 146 with Type 2 diabetes) who were selected from a large, population-based cohort (the Maastricht Study) on the basis of a moderately increased risk to develop type 2 diabetes and/or cardiovascular disease[11,16]. DNA methylation data has been measured in 188 samples collected from participants at the first follow-up evaluation of CODAM (~7 years from recruitment). A range of demographic, health and lifestyle data, serum biomarkers and clinical measures are available for these participants. Participants are primarily White Dutch, with a mean age of 65 years (range 48-79) and approximately 55% are male. DNA was derived from peripheral whole blood.

BIOS: Prospective ALS Study Netherlands (PAN)

The Prospective ALS Study Netherlands (PAN)[17] was a large-scale study of the risk factors for ALS, PSMA, PLS, Segmental and Distal SMA and PBP. Lifestyle, diet and exposure to hazardous substances are compared between patients and controls to find the risk factors. The PAN study continues from January 2020 as the Biobank Neuromuscular Diseases. In total, nearly 3,600 patients have participated in the study since its inception in 2006. We collected blood samples, cognition data and questionnaires about environmental factors, lifestyle, family history and diet from these patients.

BIOS: Illumina Infinium Methylation Assay

For the six BIOS datasets, RS, LLS, LLD, NTR, CODAM and PAN, the DNA methylation data was generated and processed identically. For the generation of genome-wide DNA

methylation data, 500 ng of genomic DNA was bisulfite modified using the EZ DNA Methylation kit (Zymo Research, Irvine, California, USA) and hybridized on Illumina 450k arrays according to the manufacturer's protocols. The original IDAT files were generated by the Illumina iScan BeadChip scanner. Data was generated by the Human Genotyping facility (HugeF) of Erasmus MC, the Netherlands (www.glimDNA.org).

BIOS: Genetic data for MR

SNPs were measured per cohort (see Ikram et al.[10] for RS, Deelen et al.[18] for LLS, Tigchelaar et al.[13] for LLD, Willemsen et al.[15] for NTR, Simons et al.[19] for CODAM and van Rheenen et al.[20] for PAN for data generation details). Genomic harmonizer[21] was used to harmonize the data, and GoNL5[22] was used as reference for imputation (Impute2[23]). SNPs were removed if they had an imputation info-score <0.5, Hardy–Weinberg equilibrium P value <10−4, call rate <95% or minor allele frequency <0.05.

BIOS: Acknowledgements

Samples were contributed by LifeLines (http://lifelines.nl/lifelines-research/general), the Leiden Longevity Study (http://www.leidenlangleven.nl), the Netherlands Twin Registry (http://www.tweelingenregister.org), the Rotterdam studies (http://www.erasmus-epidemiology.nl/research/ergo.htm), the CODAM study (http://www.carimmaastricht.nl/), and the PAN study (http://www.alsonderzoek.nl/). We thank the participants of all aforementioned biobanks and acknowledge the contributions of the investigators to this study, especially Aaron Isaacs, René Pool, Marian Beekman, P. Mila Jhamai, Michael Verbiest, H. Eka D. Suchiman, Marijn Verkerk, Ruud van der Breggen, Jeroen van Rooij, Nico Lakenberg, Jan Bot, Patrick Deelen, Irene Nooren, Martijn Vermaat, Dasha V. Zhernakova, René Luijk, Freerk van Dijk, Wibowo Arindrarto, Szymon M. Kielbasa, and Morris A. Swertz (Bios Consortium, given at the end of the paper). This work was carried out on the Dutch national e-infrastructure with the support of SURF Cooperative.

BIOS: Funding

This research was financially supported by BBMRI-NL, a Research Infrastructure financed by the Dutch government (NWO, numbers 184.021.007 and 184.033.111).

BIOS: Ethics approval and consent to participate

The study was approved by the institutional review boards of the participating centers (CODAM, Medical Ethical Committee of the Maastricht University; LL, Ethics committee of the University Medical Centre Groningen; LLS, Ethical committee of the Leiden University

Medical Center; PAN, Institutional review board of the University Medical Centre Utrecht; NTR, Central Ethics Committee on Research Involving Human Subjects of the VU University Medical Centre; RS, Institutional review board (Medical Ethics Committee) of the Erasmus Medical Center). All participants have given written informed consent and the experimental methods comply with the Helsinki Declaration.

Cardiovascular Health Study: CHS Population

The CHS is a population-based cohort study of risk factors for coronary heart disease and stroke in adults ≥65 years conducted across four field centers[24]. The original predominantly European ancestry cohort of 5,201 persons was recruited in 1989-1990 from random samples of the Medicare eligibility lists; subsequently, an additional predominantly African-American cohort of 687 persons was enrolled for a total sample of 5,888.
DNA methylation was measured on a randomly selected subset of 336 European ancestry and 329 African-American ancestry participants who participated in the 3rd annual follow-up visit (study year 5) and had DNA available from that visit. The European ancestry participants had no baseline history of coronary vascular disease (defined as coronary heart disease, congestive heart failure, peripheral vascular disease, valvular heart disease, stroke, or transient ischemic attack).

Ethic approval

CHS was approved by institutional review committees at each field centre and individuals in the present analysis had available DNA and gave informed consent including consent to use of genetic information for the study of cardiovascular disease.

DNA methylation

Methylation measurements were performed at the Institute for Translational Genomics and Population Sciences at the Harbor-UCLA Medical Center Institute for Translational Genomics and Population Sciences (Los Angeles, CA). DNA was extracted from Buffy coat fractions and subsequently underwent bisulfite conversion using the EZ DNA Methylation kit (Zymo Research, Irvine, CA). Methylation was then assayed using the Infinium HumanMethylation450 BeadChip (Illumina Inc, San Diego, CA).
Quality control was performed in in the minfi R package[25] (version 1.12.0, http://www.bioconductor.org/packages/release/bioc/html/minfi.html). Samples with low

median intensities of below 10.5 (log2) across the methylated and unmethylated channels, samples with a proportion of probes falling detection of greater than 0.5%, samples with QC probes falling greater than 3 standard deviation from the mean, sex-check mismatches, failed concordance with prior genotyping or > 0.5% of probes with a detection p-value > 0.01 were removed. Probes with >1% of values below detection were removed. In total, 11 samples were removed for sample QC resulting in a sample of 323 European-ancestry and 326 African-American samples. Methylation values were normalized using the SWAN quantile normalization method. Since white blood cell proportions were not directly measured in CHS they were estimated from the methylation data using the Houseman method.

Acknowledgements:

Infrastructure for the CHARGE Consortium is supported in part by the National Heart, Lung, and Blood Institute grant R01HL105756. The CHS research was supported by NHLBI contracts HHSN268201200036C, HHSN268200800007C, HHSN268201800001C, N01HC55222, N01HC85079, N01HC85080, N01HC85081, N01HC85082, N01HC85083, N01HC85086, R01AG023629,; 75N92021D00006 and NHLBI grants U01HL080295, U01HL130114, K08HL116640, R01HL087652, R01HL092111, R01HL103612, R01HL105756, R01HL103612, R01HL111089, R01HL116747 and R01HL120393 with additional contribution from the National Institute of Neurological Disorders and Stroke (NINDS). Additional support was provided through R01AG023629 from the National Institute on Aging (NIA), Merck Foundation / Society of Epidemiologic Research as well as Laughlin Family, Alpha Phi Foundation, and Locke Charitable Foundation. A full list of principal CHS investigators and institutions can be found at CHS-NHLBI.org. The provision of genotyping data was supported in part by the National Center for Advancing Translational Sciences, CTSI grant UL1TR000124, and the National Institute of Diabetes and Digestive and Kidney Disease Diabetes Research Center (DRC) grant DK063491 to the Southern California Diabetes Endocrinology Research Center.

The content is solely the responsibility of the authors and does not necessarily represent the official views of the National Institutes of Health.

Emory University Breast Cancer Study

The Emory University study focused on 61 Stage 0-IIIA breast cancer patients treated at Winship Cancer Institute who had received partial mastectomy with or without chemotherapy[26]. Eligible subjects were women with Stage 0-IIIA breast cancer between ages 18–75 presenting to the Winship Cancer Institute between March 2010 and November 2011. Emory Institutional Review Board approval and informed consent was obtained for all aspects of this study. For all participating patients, DNA was extracted from peripheral blood mononuclear cells, and DNA methylation was measured at >480K CpG sites via the Illumina HumanMethlation450K array. Technical replicates were included on each BeadChip and assessed for reproducibility. Further QC was performed using CpGassoc[27] to set to missing data points with probe detection p-values >0.001, and exclude CpG sites with missing data for >10% of samples. Also excluded were samples with probe detection call rates <95% and those with an average intensity value of either<50% of the experiment-wide sample mean or <2000 arbitrary units (AU). 484,489 sites remained eligible for analysis.

Outcome for CRP risk score analysis

22 (36%) of the 61 participating patients received neoadjuvant (N = 15) or adjuvant (N = 7) chemotherapy, which was completed before or after surgery, respectively, and prior to study enrollment and radiation treatment. All participating patients were treated with standard breast conserving surgery and lymph node evaluation, and all chemotherapy-treated patients received standard anthracycline- and/or taxane-based regimens. Peripheral blood sampling took place before radiation after having completed surgery and chemotherapy (if applicable); time between the last cycle of chemotherapy and blood sampling ranged from 3.7 to 18.0 weeks. Further information including exclusion criteria is provided in the parent publication.[27]

Italian cardiovascular section of EPIC (EPICOR Study)

The Italian cardiovascular section of EPIC (EPICOR study)[28] is a case-cohort study nested in the European Prospective Investigation into Cancer and Nutrition (EPIC)-Italy cohort. The EPIC-Italy cohort comprises about 50,000 participants[29] enrolled between 1992 and 1998, who provided at enrolment a detailed dietary and lifestyle questionnaire and a blood sample that was stored in liquid nitrogen for later use. The EPIC cohort is regularly followed

up for the occurrence of cancers and other non-communicable diseases of adulthood. Four EPIC-Italy centres (Turin, Varese, Naples, and Ragusa) provided samples to EPICOR. The whole EPICOR study comprises more than 1,500 subjects with cardiovascular outcomes such as myocardial infarction (MI), acute coronary syndrome, ischemic cardiomyopathy, coronary or carotid revascularization, ischemic- or haemorrhagic stroke. Within the EPICOR cohort, a subset of 584 subjects (292 MI cases and 292 matched controls) was analysed as a nested case-control study and underwent DNA methylation analysis and whole genome genotyping. All volunteers signed an informed consent form at enrolment in the respective studies. EPICOR study complies with the Declaration of Helsinki principles and conforms to ethical requirements

Ethic approval

The EPIC study protocol was approved by Ethics Committees of the International Agency for Research on Cancer (Lyon, France), as well as by local Ethical Committees of the participant centres. The EPICOR study was approved by the Ethical Committee of the Italian Institute for Genomic Medicine (IIGM, formerly Human Genetics Foundation-Torino, HuGeF, Turin, Italy).

Methylation measurements

DNA methylation was measured in DNA from WBCs collected at subject enrolment into EPIC and stored in liquid nitrogen. Genomic DNA was extracted from 400ul buffy coat from whole blood stored in liquid nitrogen at sample recruitment by an automated on-column DNA purification method (QIAsymphony instrument and QIAsymphony DNA Kits, QIAGEN GmbH, Germany), according to manufacturer’s standard protocols. DNA integrity was checked by an electrophoretic run in standard TBE 0.5X buffer on a 1% low melting agarose gel (Sigma-Aldrich GmbH, Germany); DNA purity and concentration were assessed by a NanoDrop 8000 Spectrophotometer (Thermo Fisher Scientific Inc.). Five hundreds of genomic DNA were bisulphite converted (EZ-96 DNA Methylation-Gold Kit, Zymo Research Corporation) according to manufacturer’s protocol. The methylation status of more than 485,000 individual CpG loci at a genome-wide resolution was assessed by the Infinium HumanMethylation450 BeadChip (Illumina Inc., San Diego, CA, USA) according to standard manufacturer protocols. Functional normalization for Whole-genome methylation data quality control (QC) and normalization procedures was performed: a total of 292 matched case-control pairs (584 subjects) passed QCs and 484683 CpG sites passed QCs and were retained for further analyses. Potential confounding effects of blood cell subtypes were

estimated by the Houseman method. To account for batch effects in the data, beta values underwent a functional normalization approach using the first 20 PCs of the Illumina 450K array control probes.

ESTHER cohort

The ESTHER cohort, as previously described in detail[30], is an ongoing population-based cohort study conducted in Saarland, Germany. 9,940 older participants (age 50-75 years) were recruited by their general practitioners during routine health check-ups between 2000 and 2002. During baseline enrolment, information on demographic characteristics and lifestyle variables were obtained from a standardized self-administered questionnaire and biological samples (blood, stool and urine) were collected. Comprehensive medical data, medical diagnoses and drug prescriptions were additionally obtained from the general practitioner. Genome-wide DNA methylation measurements of the ESTHER Study were performed in the baseline blood samples of two subsets with non-overlapping sets of participants. ESTHER-1a consists of 1,000 participants who were recruited between July and October 2000 and ESTHER-1b consists of 548 participants who were recruited between October 2000 and March 2001. After excluding participants without CRP test data, 974 and 543 participants were left in ESTHER-1a and ESTHER-1b, respectively.

Conflicts of Interest

HJG has received travel grants and speakers honoraria from Fresenius Medical Care, Neuraxpharm, Servier and Janssen Cilag as well as research funding from Fresenius Medical Care.

Estonian Biobank (EstBB)

The Estonian Biobank is a population-based biobank of the Estonian Genome Centre at the University of Tartu (EstBB) Leitsalu et al. 2015[31]. The entire project is conducted according to the Human Genes Research Act of Estonia and all of the participants have signed the broad informed consent. The cohort size is currently close to 200,000 participants aged ≥18, which closely reflects the age, sex and geographical distribution of the Estonian population. The samples used in this study were selected from the EstBB Center for Translational Genomics (CTG) cohort of individuals who have been recontacted for a second time-point

sample (EstBB-CTG). DNA methylation was measured from whole blood with the Illumina 450K array. Data was normalized according to the CPACOR pipeline[32]. Probes with >5% of samples having a detection P-value of > 1e-16 were excluded and samples with 95% of probes have a detection P-value of < 1e-16 were retained. DNA methylation data from 306 samples and 470,220 probes were used for the analyses.

Acknowledgements and Funding

The research of the EstBB cohort was supported by the European Union through the European Regional Development Fund (Project No. 2014-2020.4.01.15-0012). Data analyses was carried out in part in the High-Performance Computing Center of University of Tartu.

Estonian Biobank Research Team: group author acknowledgement

Tõnu Esko, Andres Metspalu, Reedik Mägi, Mari Nelis

Kooperative Gesundheitsforschung in der Region Augsburg (KORA) F4

The KORA (Kooperative Gesundheitsforschung in der Region Augsburg) study[33] has been collecting clinical and genetic data from the general population in the region of Augsburg, Germany for more than 20 years. The cohort investigated in this paper is the F4 study (2006-2008), a follow-up of the S4 study (1999-2001). The participants completed a questionnaire and underwent standardized examinations with blood samples taken, as described elsewhere[33,34].

DNA methylation data measurement:

Genome-wide DNA methylation measurement was performed in whole blood using the Infinium HumanMethylation450K BeadChip in 1802 KORA F4 samples, with laboratory process as described previously[35]. DNA methylation data were preprocessed following the CPACOR pipeline[32]. Following removal of the 65 probes representing SNPs and background correction (R package minfi, v1.6.0)[25], probes with detection p-value ≥ 0.01 or summarized by < 3 functional bead were removed. Observations with >5% missing values were excluded, resulting in 1727 samples overall.

To reduce the non-biological variability between observations, quantile normalization was performed on a stratification of the probe categories into 6 types, based on probe type and color channel (R package limma, v3.16.5) (Smyth, 2005). To further reduce technical

variation, the first 30 principal components of the non-negative methylation control probes were used as covariates in the regression models, as were proportions of white blood cell types (granulocytes, monocytes, B cells, CD4+ T cells, CD8+ T cells and natural killer cells) estimated using the procedure of Houseman et al.[36]

Outcome for CRP risk score analysis

For the diagnosis of prevalent type II diabetes, prevalent coronary artery disease and previous myocardial infarction, self-report was used at the time of the interview. Hypertension was defined systolic blood pressure > 140mmHg, or DBP > 90mmHg, or intake of anti-hypertensive or blood pressure-lowering medication.

Genetic data for MR:

After performing standard sample QC we included 3,788 individuals from KORA that were genotyped on the AffyAxiom array. 558,446 variants were included in the imputation scaffold. Variants were imputed to the HRC reference r1.1 2016 on the Michigan Imputation Server.

Acknowledgements

The KORA study was initiated and financed by the Helmholtz Zentrum München –German Research Center for Environmental Health, which is funded by the German Federal Ministry of Education and Research (BMBF) and by the State of Bavaria. Furthermore, KORA research was supported within the Munich Center of Health Sciences (MC-Health), Ludwig-Maximilians-Universität, as part of LMUinnovativ.

Lothian Birth Cohorts (LBC1936)

The Lothian Birth Cohorts of 1936 is a longitudinal study of ageing[37-40]. It derives from the Scottish Mental Survey of 1947 when nearly all 11 year old children in Scotland completed a test of general cognitive ability[39]. Survivors living in the Lothian area of Scotland were recruited in late-life at mean age 70 (n=1,091). Follow-up has taken place triennially. Collected data include genetic information, longitudinal epigenetic information, longitudinal brain imaging, and numerous blood biomarkers, anthropomorphic and lifestyle measures.

DNA methylation:

Detailed information about the collection and QC steps undertaken on the LBC methylation data have been reported previously[41]. Briefly, the Infinium HumanMethylation450 BeadChip

(Illumina Inc, San Diego, CA) was used to measure DNA methylation in whole blood of consenting participants. Background correction was performed and QC was used to remove probes with a low detection rate, low quality (manual inspection), low call rate, and samples with a poor match between genotypes and SNP control probes, and incorrect predicted sex. At the second LBC1936 visit, non-fasting blood samples were collected. CRP levels were measured by a high-sensitivity assay at the University of Glasgow using an enzyme-linked immunosorbent assay (ELISA; R&D Systems, Oxford, UK). Post QC, DNA methylation data and CRP levels were available at 459,329 CpG sites for 258 participants.

At each wave of the respective studies, basic anthropometric measures were taken, including height and weight. Body mass index was calculated as weight in kilogram divided by height in metres squared. White blood cell counts (eosinophils, basophils, neutrophils, lymphocytes, and monocytes) were also measured at each wave[42].

Data Availability

LBC data are available on request from the Lothian Birth Cohort Study, University of Edinburgh (Simon Cox, simon.cox@ed.ac.uk). LBC data are not publicly available due to them containing information that could compromise participant consent and confidentiality.

Acknowledgements

The LBC1936 is supported by Age UK (Disconnected Mind program) and the Medical Research Council (MR/M01311/1). Methylation typing was supported by Centre for Cognitive Ageing and Cognitive Epidemiology (Pilot Fund award), Age UK, The Wellcome Trust Institutional Strategic Support Fund, The University of Edinburgh, and The University of Queensland.

LOLIPOP:

LOLIPOP is a prospective cohort study of ~28K Indian Asian and European men and women, recruited from the lists of 58 General Practitioners in West London, United Kingdom between 2003 and 2008. At enrolment all participants completed a structured assessment of cardiovascular and metabolic health, including anthropometry, and collection of blood samples for measurement of fasting glucose, insulin and lipid profile, HbA1c, and complete blood count with differential white cell count. Aliquots of whole blood were stored at -80C

for extraction of genomic DNA. Epigenome-wide association was performed using genomic DNA from peripheral blood collected at enrolment. The LOLIPOP study is approved by the National Research Ethics Service (07/H0712/150) and all participants gave written informed consent.

Acknowledgements and Funding

LOLIPOP study was funded by the National Institute for Health Research (NIHR) (16/136/68) using UK aid from the UK Government to support global health research, and by Wellcome Trust (212945/Z/18/Z). The views expressed in this publication are those of the author(s) and not necessarily those of the NIHR or the UK Department of Health and Social Care. John Chambers is supported by the Singapore Ministry of Health's National Medical Research Council under its Singapore Translational Research (STaR) Investigator (NMRC/STaR/0028/2017).

Northern Finland Birth Cohort 1966 (NFBC1966)

The Northern Finland Birth Cohort 1966 is a prospective follow-up study of children from the two northernmost provinces of Finland [43] 96% of all woman in this region with expected delivery dates in 1966 were recruited though maternity health centres (12,058 live births). All individuals still living in northern Finland or the Helsinki area (*n* = 8,463) were contacted and invited for clinical examination. A total of 6007 participants attended the clinical examination at the participants' age of 31 years. DNA was extracted from blood samples given at the clinical examination (5,753 samples available) [44].The subset with DNA is representative of the original cohort in terms the major environmental and social factors known to influence the tested trait. An informed consent for the use of the data including DNA was obtained from all subjects. DNA methylation was measured for 807 randomly selected subjects that attended the clinical examination and completed the questionnaire For DNA methylation marker calling we used a detection P-values threshold of $<10^{-16}$ A call rate filter of 95% was applied to the all autosomal Illumina probes yielding 459378 probes for association testing. 67 samples were excluded due to low marker call rate (<95%). 7 samples were excluded for gender inconsistency, one sample for globally outlying DNA methylation values (1st PC score of the DNA methylation values outside mean +/- 4SD).

Genetic data for MR:

In NFBC1966 a total of 5,402 NFBC1966 participants were genotyped on an Illumina HumanCNV370DUO Analysis BeadChip. 329,401 variants were included in the imputation scaffold. Variants were imputed to the HRC reference r1.1 2016 on the Michigan Imputation Server. For Mendelian Randomization analysis we restricted the dataset to participants with DNA methylation data available (n=706).

Outcome forCRP risk score analysis

In NFBC1966, we used a Vitalograph P-model spirometer (Vitalograph Ltd., Buckingham, UK), with a volumetric accuracy of ±2% or ±50 mL whichever was greater. The spirometer was calibrated regularly using a 1-Litre precision syringe. The spirometric manoeuvre was performed three times but was repeated if the coefficient of variation between two maximal readings was >4. Participants with values below the lower limit of normal as defined by Global Lung Intiative (GLI) were coded as COPD cases. Type 2 diabetes in NFBC1966 was defined as either or: prescription of metformin (Finnish register for reimbursed medication; ATC code A10B, available from year 1997 and 2016), diagnosed by a physician (Finnish outpatient register; ACD9 or ICD10 code E11*) or screen-detected by OGTT at the age of 46y (NFBC1966 clinical follow-up in 2012)

Acknowledgements:

We thank all cohort members and researchers who participated in the 31y and 46y NFBC1966 study. We also wish to acknowledge the work of the NFBC project centre. NFBC1966 received financial support from University of Oulu Grant no. 65354, Oulu University Hospital Grant no. 2/97, 8/97, Ministry of Health and Social Affairs Grant no. 23/251/97, 160/97, 190/97, National Institute for Health and Welfare, Helsinki Grant no. 54121, Regional Institute of Occupational Health, Oulu, Finland Grant no. 50621, 54231, University of Oulu Grant no. 24000692, Oulu University Hospital Grant no. 24301140, ERDF European Regional Development Fund Grant no. 539/2010 A31592, Academy of Finland grant numbers 24300796, 24302031, 285547 (EGEA) (MRJ); the Medical Research Council (MRC) UK (grant number G0601653) (MRJ); Medical Research Council Biotechnology and Biological Sciences Research Council *PREcisE* (Nutrition & Epigenome, The Joint Programming Initiative a Healthy Diet for a Healthy Life (JPI HDHL/EU-H2020)) (MRJ); Yrjö Jahnsson Foundation (SP), Päivikki and Sakari Sohlberg Foundation sr (SP); the European Union's Horizon 2020 programmes, iHealth-T2D (grant number 643774) and EDCMET (grant number 825762) (SP).

Northern Finland Birth Cohort 1986 (NFBC1986)

The Northern Finland Birth Cohort 1986 consists of 99% of all children, who were born in the provinces of Oulu and Lapland in Northern Finland between 1 July 1985 and 30 June 1986. 9,203 live-born individuals entered the study[44]. At the age of 16, the subjects living in the original target area or in the capital area (n=9,215) were invited to participate in a follow-up study including a clinical examination. 7344 participants attend the study in year 2001/2002, of which 5654 completed the postal questionnaire, the clinical examination and provided a blood sample. DNA was extracted from all 5654 blood samples. An informed consent for the use of the data including DNA was obtained from all subjects. DNA methylation was recoded on Illumina HumanMethlation450K array for 566 randomly selected subjects. 24 technical replicates were excluded. 18 samples did not reach a call rate of >95% applying a detection P-value filter of 10^{-16}. We excluded 7 samples with gender inconsistency, no sample was outlying from the overall data structure (1st PC score of the DNA methylation values outside mean +/- 4SD). DNA methylation data of 517 samples with 466290 autosomal probes (call rate filter 95%) each were used for this analysis.

Genetic data for MR:

After performing standard sample QC we included 3,743 NFBC1986 participants that were genotyped on an Illumina Human Omni Express Exome 8v1.2 BeadChip. 889,119 variants were included in the imputation scaffold. Variants were imputed to the HRC reference r1.1 2016 on the Michigan Imputation Server.

Acknowledgements:

We thank all cohort members and researchers who have participated in the NFBC1986 study. We also wish to acknowledge the work of the NFBC project center. NFBC1986 received financial support from EU QLG1-CT-2000-01643 (EUROBLCS) Grant no. E51560, NorFA Grant no. 731, 20056, 30167, USA / NIH 2000 G DF682 Grant no. 50945.

Data sharing:

NFBC data is available from the University of Oulu, Infrastructure for Population Studies. Permission to use the data can be applied for research purposes via electronic material request portal. In the use of data, we follow the EU general data protection regulation (679/2016) and Finnish Data Protection Act. The use of personal data is based on cohort

participant's written informed consent at his/her latest follow-up study, which may cause limitations to its use. Please, contact NFBC project center (NFBCprojectcenter@oulu.fi) and visit the cohort website (www.oulu.fi/nfbc) for more information.

Rotterdam Study (RS)

Rotterdam Study (RS) is a prospective population-based cohort study in a well-defined area of Rotterdam, the Netherlands. General design and overview of the study can be found described in more details elsewhere [45]. For the current analysis we used data from individuals aged 45 years and older that participated in the third cohort of the Rotterdam Study (RS-III). In the first visit of the third cohort (RS-III-1), 3,934 participants were examined between February 2006 and December 2008.

DNA methylation

Whole blood DNA methylation was quantified in a random subset of ~750 individuals with genotyping and RNA expression data available. DNA was extracted from whole peripheral blood (stored in EDTA tubes) by standardized salting out methods. Genome-wide DNA-methylation levels in ~750 subjects were determined using the Illumina HumanMethylation 450K beadarray (Illumina, Inc., San Diego, CA, USA). In short, samples (500ng of DNA per sample) were first bisulfite treated using the Zymo EZ-96 DNA-methylation kit (Zymo Research, Irvine, CA, USA). Next, they were hybridized to the arrays according to the manufacturers protocol. During quality control samples showing incomplete bisulfite treatment were excluded (n=5) as were samples with a low detection rate (0.01 in >1% samples, were filtered out. A total number of 474,528 probes passed the quality control and the filtered β values were normalized with DASEN implemented in the wateRmelon package in R statistical software. At the first center visit, fasting blood samples were collected. The samples were immediately put on ice and were processed within 30 minutes after which the samples were kept frozen at -80 °C until the measurement of high-sensitivity CRP (hs-CRP) in January 2012. Serum CRP was measured by a particle enhanced immunoturbidimetric assay (Roche Diagnostics GmnH, Mannheim, Germany). This assay measures CRP values ranging from 0.3-350 mg/L. From the 734 available methylation samples, after excluding individuals with auto-immune diseases and individuals using immune-modulating agents, the total number of participants with serum CRP levels and DNA methylation measurement was 722.

During the research center visit, anthropometric measures including height and weight were obtained. Body mass index was calculated as weight in kilogram divided by height in meters squared. Smoking behavior (current, former and never) was assessed during home interview by trained research assistants. White blood cells counts (monocytes, granulocytes and lymphocytes) were measured immediately at the research center using a standard hematology analyzer (Beckman Coulter, Pasadena, CA, USA).

The Study of Health in Pomerania (SHIP-Trend)

The Study of Health in Pomerania is a longitudinal population-based cohort study in West Pomerania, a region in the northeast of Germany, assessing the prevalence and incidence of common population-relevant diseases and their risk factors. Baseline examinations for SHIP-Trend were carried out between 2008 and 2012, comprising 4,420 participants aged 20 to 81 years. Study design and sampling methods were previously described Völzke, H. et al.[46] The medical ethics committee of the University of Greifswald approved the study protocol, and oral and written informed consents were obtained from each of the study participants.

DNA methylation

DNA was extracted from blood samples of n=256 SHIP-Trend participants to assess DNA methylation using the Illumina HumanMethylationEPIC BeadChip array. Samples were randomly selected based on availability of multiple OMICS data, excluding type II diabetes, and enriched for prevalent MI. The samples were taken between 07:00 AM and 04:00 PM, and serum aliquots were prepared for immediate analysis and for storage at -80 °C in the Integrated Research Biobank (Liconic, Liechtenstein). Processing of the DNA samples was performed at the Helmholtz Zentrum München. Preparation and normalization of the array data was performed according to the CPACOR workflow[32] using the software package R (www.r-project.org). Arrays with observed technical problems (±4SD outside control probe intensity mean) during steps like bisulfite conversion, hybridization or extension, as well as arrays with mismatch between sex of the proband and sex determined by the chr X and Y probe intensities were removed from subsequent analyses.

Details on assessment of the phenotypes and covariates used in this analysis are provided within the SHIP cohort design paper.

Acknowledgements

SHIP is part of the Community Medicine Research net of the University of Greifswald, Germany, which is funded by the Federal Ministry of Education and Research (grants no. 01ZZ9603, 01ZZ0103, and 01ZZ0403), the Ministry of Cultural Affairs as well as the Social Ministry of the Federal State of Mecklenburg-West Pomerania, and the network 'Greifswald Approach to Individualized Medicine (GANI_MED)' funded by the Federal Ministry of Education and Research (grant 03IS2061A). DNA methylation data have been supported by the DZHK (grant 81X3400104). The University of Greifswald is a member of the Caché Campus program of the InterSystems GmbH. The SHIP authors are grateful to Paul S. DeVries for his support with the EWAS pipeline.

TwinsUK Cohort

The TwinsUK cohort was established in 1992 and comprises adult same-sex monozygotic and dizygotic twins in the UK. The cohort has over 14,000 registered volunteer twins[47]. In this study, we included 416 female participants who had both blood DNA methylation profiles and serum CRP levels measured. DNA extracted from whole blood samples stored in EDTA tubes was used for DNA methylation profiling. Infinium HumanMethylation450 BeadChip (Illumina) was used to assess blood DNA methylation. Details of DNA extraction and methylation measurement are described by Tsaprouni et al.[48].Quantile normalization was used to minimize the technical variation arising from the design of the two Illumina probes. Probes with incorrect or non-exclusive mapping of DNA methylation signals to reference sequences were excluded. Signals with detection P values > 1×10-16 were assigned as missing data. Probes were removed if more than 5% of all samples were missing. Subjects with abnormal overall methylation distribution or missing methylation probes > 5% were removed. After quality control, a total of 473,864 probes were included for further analysis. A linear mixed effect regression model was applied to each methylation probe to detect the association between DNA methylation levels and natural log transformed CRP values. Family structure and zygosity were included as random effect terms in the model, and the other covariates, such as age, BMI, imputed white blood cell counts, and 10 control probe PCs were included as fixed effect terms. Ethical approval was obtained from the London-Westminster National Research Ethics Service, St Thomas'

Hospital Research Ethics Committee (EC04/015 and 07/H0802/84). All twins provided written informed consent prior to participation in the study.

Acknowledgements

TwinsUK is funded by the Wellcome Trust (grants WT081878MA and WT202786/Z/16/Z contributed to the majority of data described), as well as the Medical Research Council, European Union, the National Institute for Health Research (NIHR)-funded BioResource, Clinical Research Facility and Biomedical Research Centre based at Guy's and St Thomas' NHS Foundation Trust in partnership with King's College London.

This project also received support from the JPI ERA-HDHL DIMENSION project and UK Biological Sciences Research Council (BBSRC, BB/S020845/1 and BB/T019980/1 to JTB). P-C Tsai was funded by Chang Gung Memorial Hospital, grant number CMRPD1J0082 /Ministry of Science and Technology, Taiwan, grant number NMRPD1K0941 and NZRPD1K0011.

The Young Finns Study (YFS)

The Cardiovascular Risk in Young Finns Study is an on-going multicentre follow-up study of atherosclerosis precursors of Finnish children and adolescents. The first cross-sectional survey was conducted in 1980. Total sample size was 4,320 children and adolescents aged 3, 6, 9, 12, 15 and 18 years. The subjects were randomly chosen from the national register. Total of 3,596 subjects (83.2 percent of those invited) participated in 1980. Follow-up studies have been conducted in 1983, 1986, 2001, 2007, 2011, 2018-2019 with the original cohort.

DNA methylation

Leukocyte DNA of the YFS cohort from 2011 follow-up was obtained from EDTA-blood samples using a Wizard® Genomic DNA Purification Kit (Promega Corporation, Madison, WI, USA) according to the manufacturer's instructions. Genome-wide DNA methylation levels were obtained using Illumina Infinium HumanMethylation 450k BeadChip and Infinium MethylationEPIC array according to the protocol by Illumina. All the analysed samples have sum of detection P-values across all the probes less than 0.01. Logged (log2) median of methylated and unmethylated intensities of the analysed samples clustered visually well. Further, samples for which real sex did not match the predicted sex were excluded. Background subtraction and dye-bias normalization was performed via noob method[49]

followed by stratified quantile normalization. Probes with detection p-value more than 0.01 in 99% of the samples were filtered out. All the pre-processing steps were performed using functions implemented in minfi R/Bioconductor package[25].

Acknowledgements

"The Young Finns Study has been financially supported by the Academy of Finland: grants 322098, 286284, 134309 (Eye), 126925, 121584, 124282, 129378 (Salve), 117787 (Gendi), and 41071 (Skidi); the Social Insurance Institution of Finland; Competitive State Research Financing of the Expert Responsibility area of Kuopio, Tampere and Turku University Hospitals (grant X51001); Juho Vainio Foundation; Paavo Nurmi Foundation; Finnish Foundation for Cardiovascular Research; The Sigrid Juselius Foundation; Tampere Tuberculosis Foundation; Emil Aaltonen Foundation; Yrjö Jahnsson Foundation; Signe and Ane Gyllenberg Foundation; Diabetes Research Foundation of Finnish Diabetes Association; EU Horizon 2020 (grant 755320 for TAXINOMISIS); This project has received funding from the European Union’s Horizon 2020 research and innovation programme under grant agreement No 848146 (To Aition); European Research Council (grant 742927 for MULTIEPIGEN project); Tampere University Hospital Supporting Foundation and Finnish Society of Clinical Chemistry."

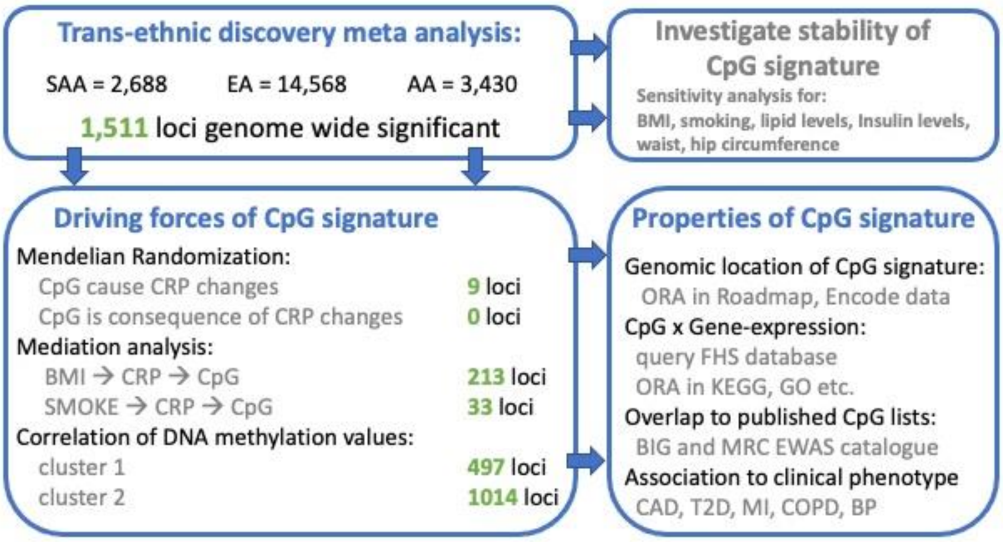

Supplementary Results:

Supplementary figure 1: Study overview. Flow of analysis starts with trans-ethnic discovery. Followed by analysis presented in the manuscript. ORA is short for over representation analysis (see online method section Overrepresentation analysis). Description of the Roadmap project incl. their publicly available data sets are available on their project homepage (http://www.roadmapepigenomics.org/data/)FHS database indicates results from CpG x Gene expression analysis in Framingham heart study relevant for this study. BIG EWAS catalogue is supplied by National Genomics Datacenter China (https://bigd.big.ac.cn/databasecommons/database/id/6285) MRC EWAS catalogue is provided by the University of Bristol (http://www.ewascatalog.org)

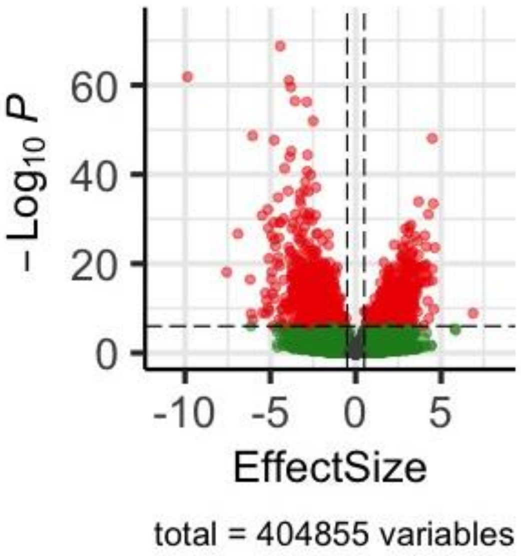

Supplemental Figure 2: Volcano plot CpG methylation and serum CRP association result. Red dots were taken forward to further analysis in the presented study. Each dot represents one P-value from transethnic discovery analysis (described in more detail in method section Cohort-specific CRP DNA methylation associations" and "Meta-Analysis and Genomic control procedure")

Dotted horizontal line is P-value threshold 1xE-07. Vertical line indicates smallest observed effect size of CpG in analysis. Effect size is logarithmic ml/L change in CRP per unit increase in DNA methylation.

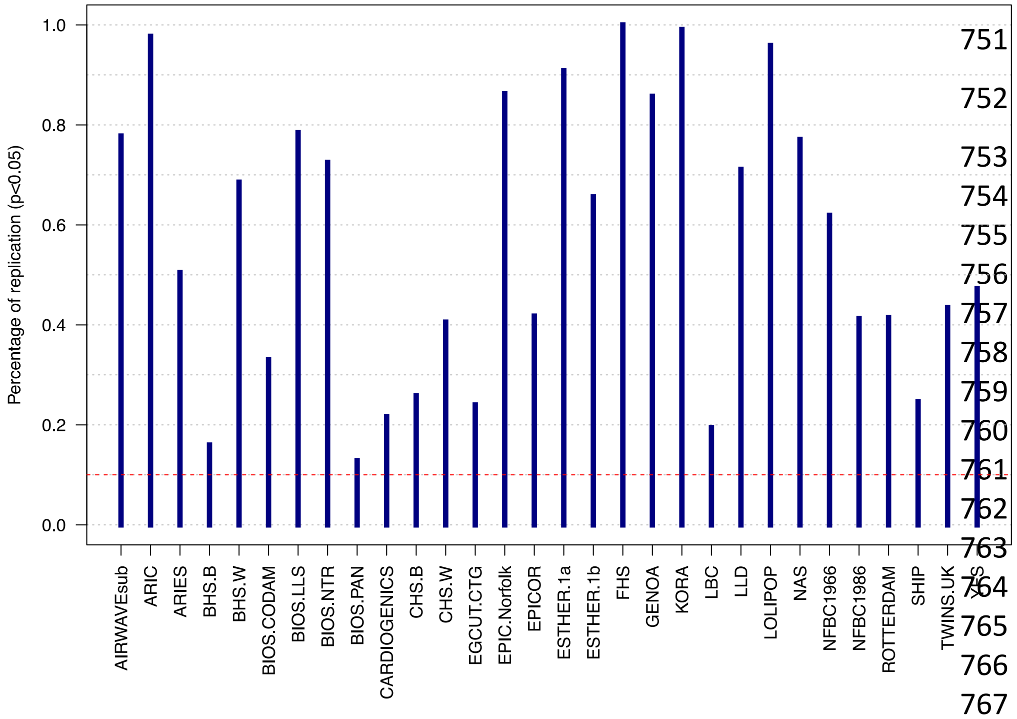

Supplementary Figure 3: Proportion of replicated CpGs. 218 CpG sites identified in Ligthart et al. Genome Biology (2016) were measured in each of the participating cohorts in the present study. Successful replication for any of the 218 markers in each cohort was defined as P value below 0.05 for the association between CpG methylation and serum CRP levels (described in more detail in method section Cohort-specific CRP DNA methylation associations).

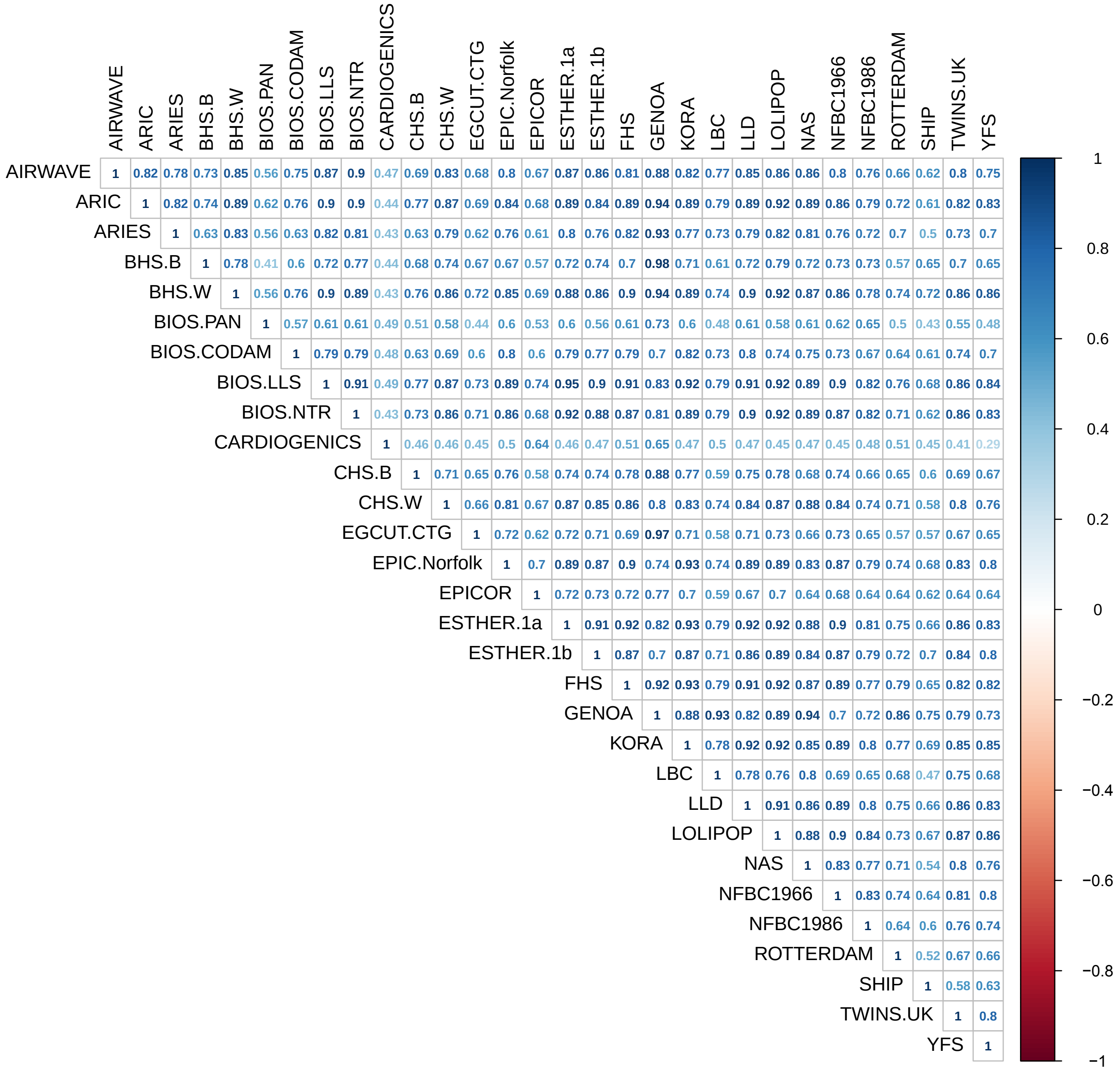

Supplementary Figure 4: Correlation of effect estimates for CRP association. As with Supplemental Figure 2 we restricted the analysis to 218 CpG sites reported as associated with blood CRP levels in Ligthart et al. We calculated Pearson correlation coefficients of pairwise complete observation between the cohorts (described in more detail in method section Correlation within CRP-associated markers).

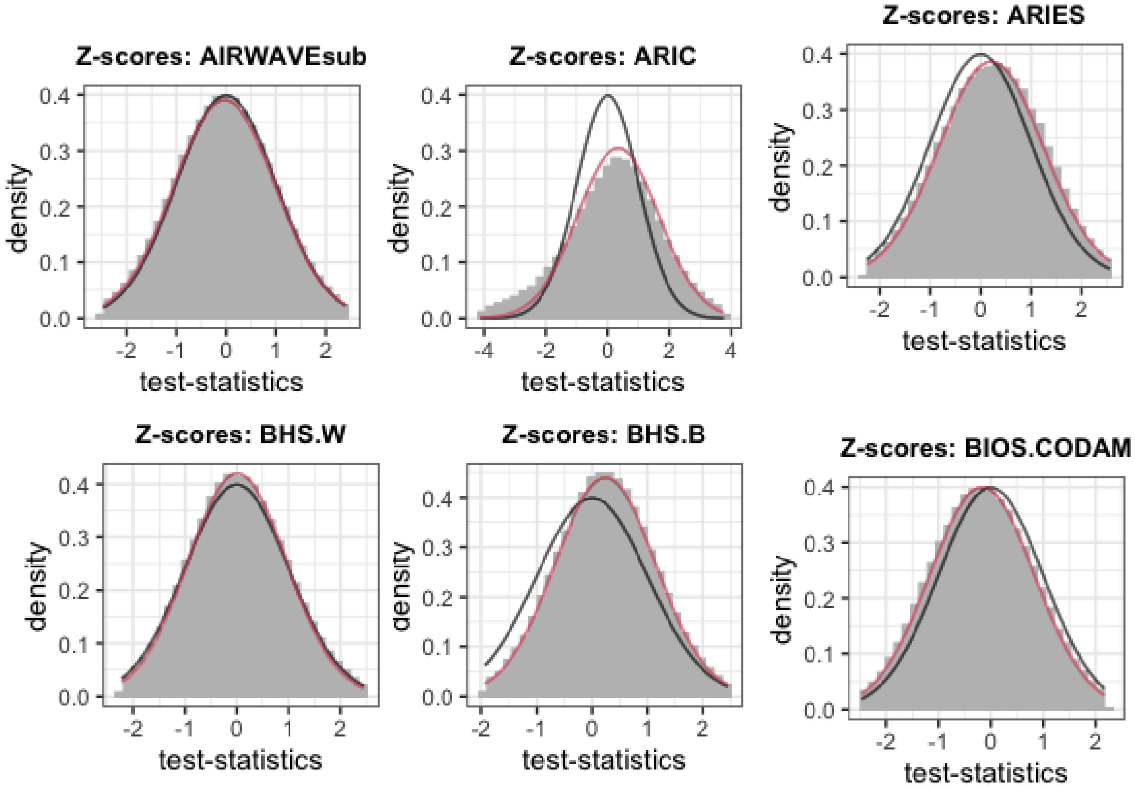
Z-scores: AIRWAVEsub
Z-scores: ARIC
Z-scores: ARIES
Z-scores: BHS.W
Z-scores: BHS.B
Z-scores: BIOS.CODAM
density
test-statistics

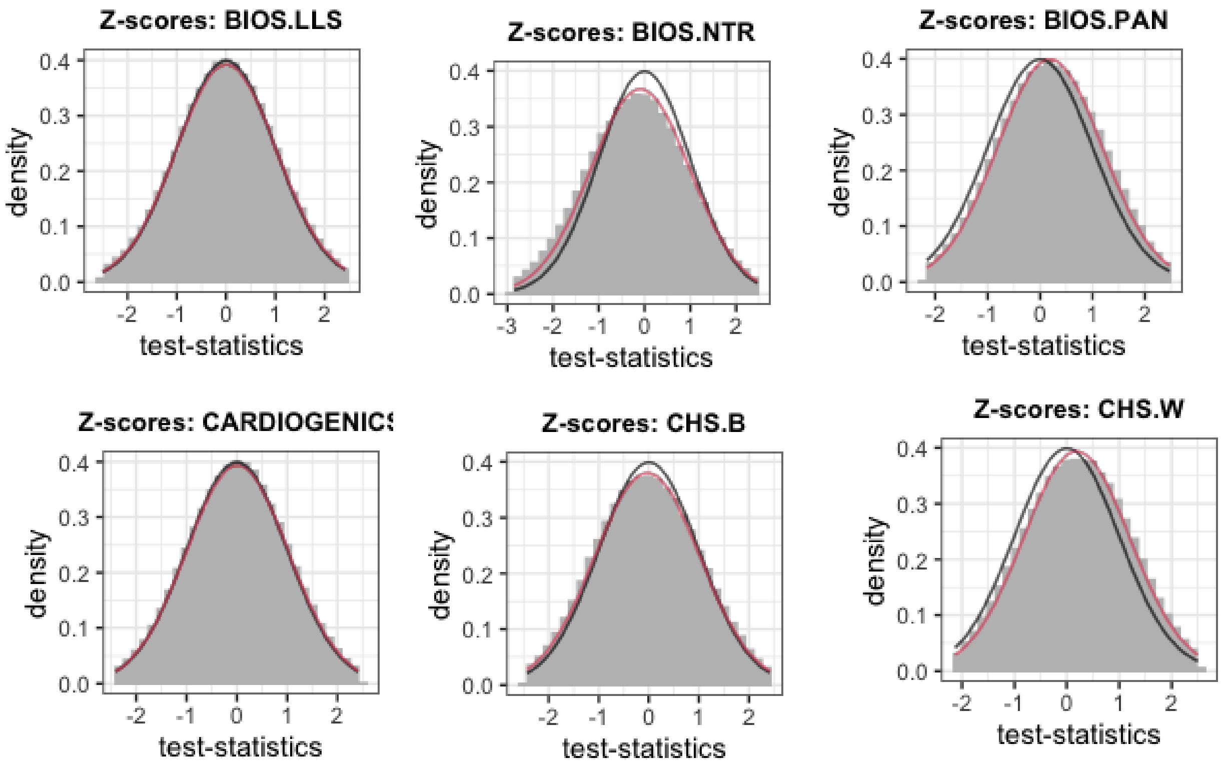
Z-scores: BIOS.LLS
Z-scores: BIOS.NTR
Z-scores: BIOS.PAN
Z-scores: CARDIOGENICS
Z-scores: CHS.B
Z-scores: CHS.W
density
test-statistics

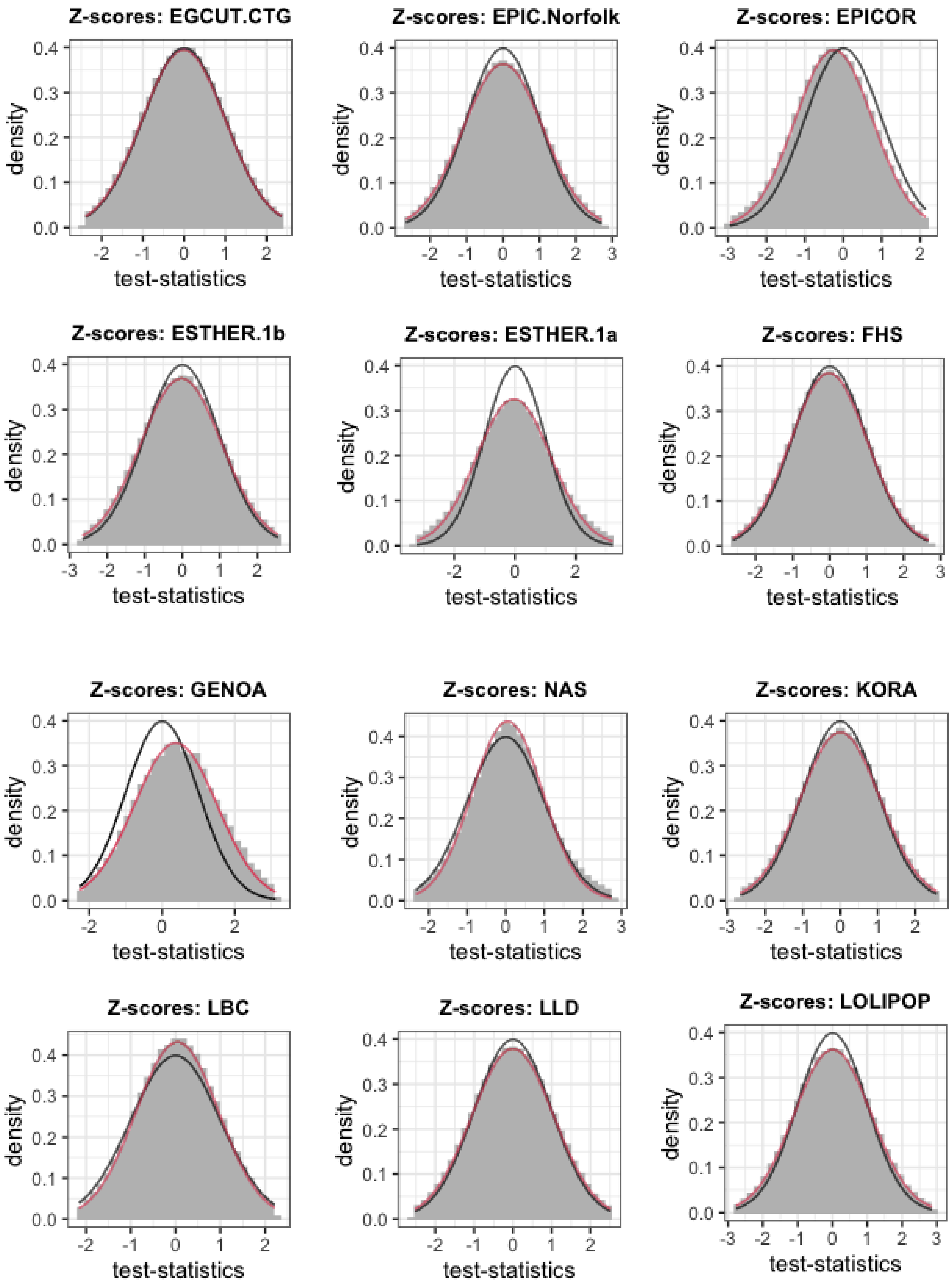
Z-scores: EGCUT.CTG
Z-scores: EPIC.Norfolk
Z-scores: EPICOR
Z-scores: ESTHER.1b
Z-scores: ESTHER.1a
Z-scores: FHS
Z-scores: GENOA
Z-scores: NAS
Z-scores: KORA
Z-scores: LBC
Z-scores: LLD
Z-scores: LOLIPOP
density
test-statistics

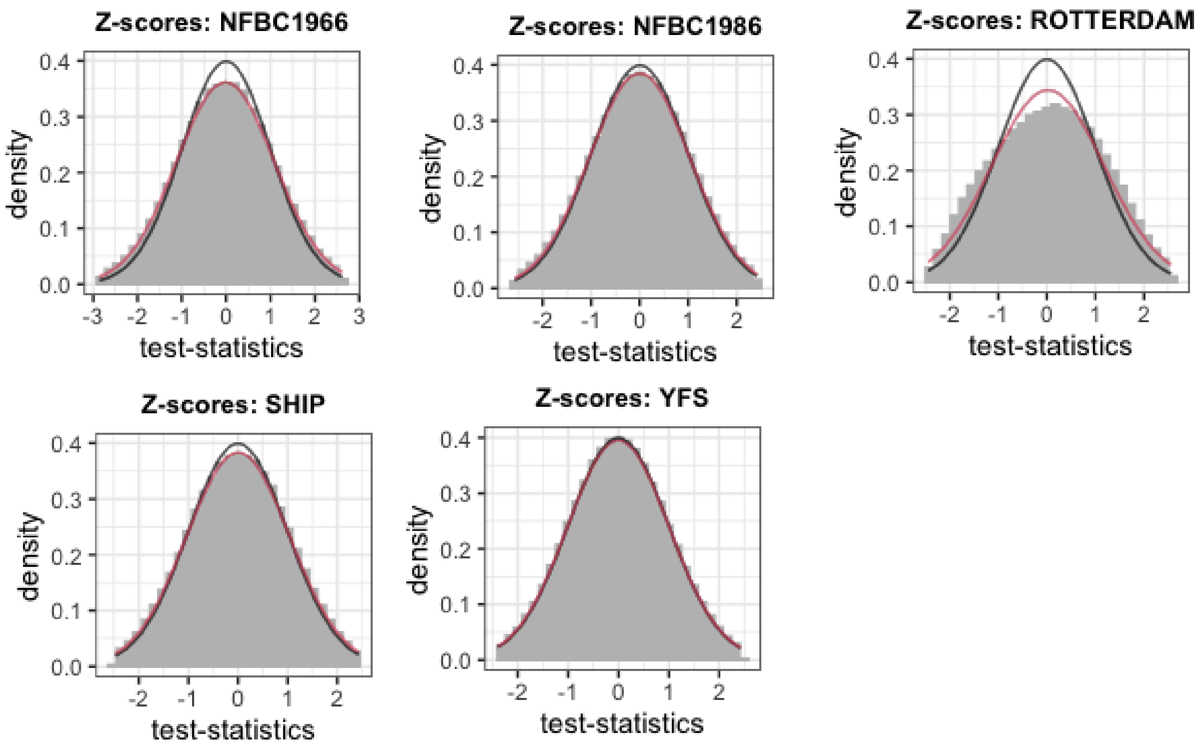

Supplemental Figure 5: Evaluation of test statistic bias from individual logistic regression analysis (method section: Cohort-specific CRP DNA methylation associations). Z-scores were calculated by dividing the effect estimate by the standard error (as implemented in R-package BACON). We inspected the distribution of each the test statistics contributing to our transethnic meta-analysis to evaluate their deviation from the empirical null distribution. Black line represents empirical null distribution. Red line represents observed distribution. The majority of studies did not deviate from empirical null distribution

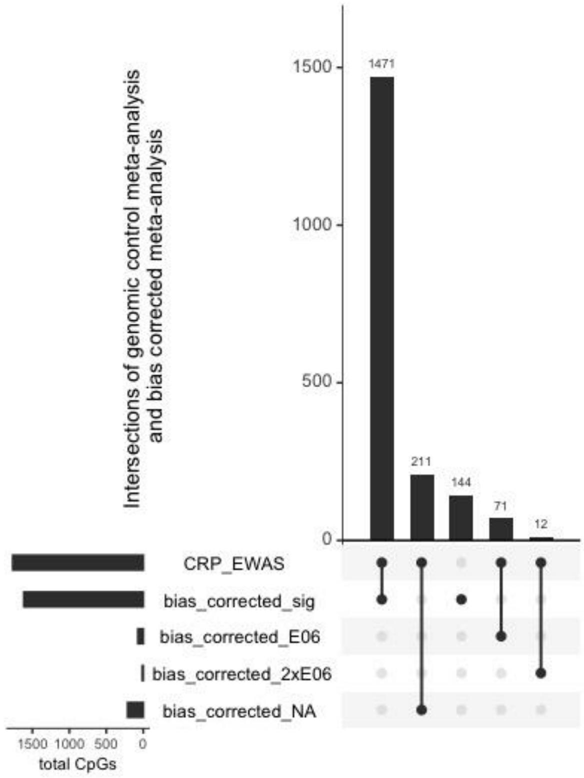

Supplementary Figure 6: Upset plot shows the overlaps between meta-analysis using genomic control and meta-analysis using test-statistic bias correction method (see method section Cohort-specific CRP DNA methylation associations). CRP EWAS is the complete lists of all markers presented in the study (n=1765). This includes correlated markers, which were removed from downstream analysis in the manuscript. “bias_corrected_sig” is the list of markers with a P value smaller than 1xE-07 from bias corrected method. “bias_corrected_E06” are all markers, significant in genomic control meta-analysis and showing a P value of smaller than 1xE-06 in bias corrected meta-analysis. “bias_corrected_2xE06” same as above with threshold relaxed to 2xE-06. “bias_corrected_NA” are marker not available in current bias corrected meta-analysis.

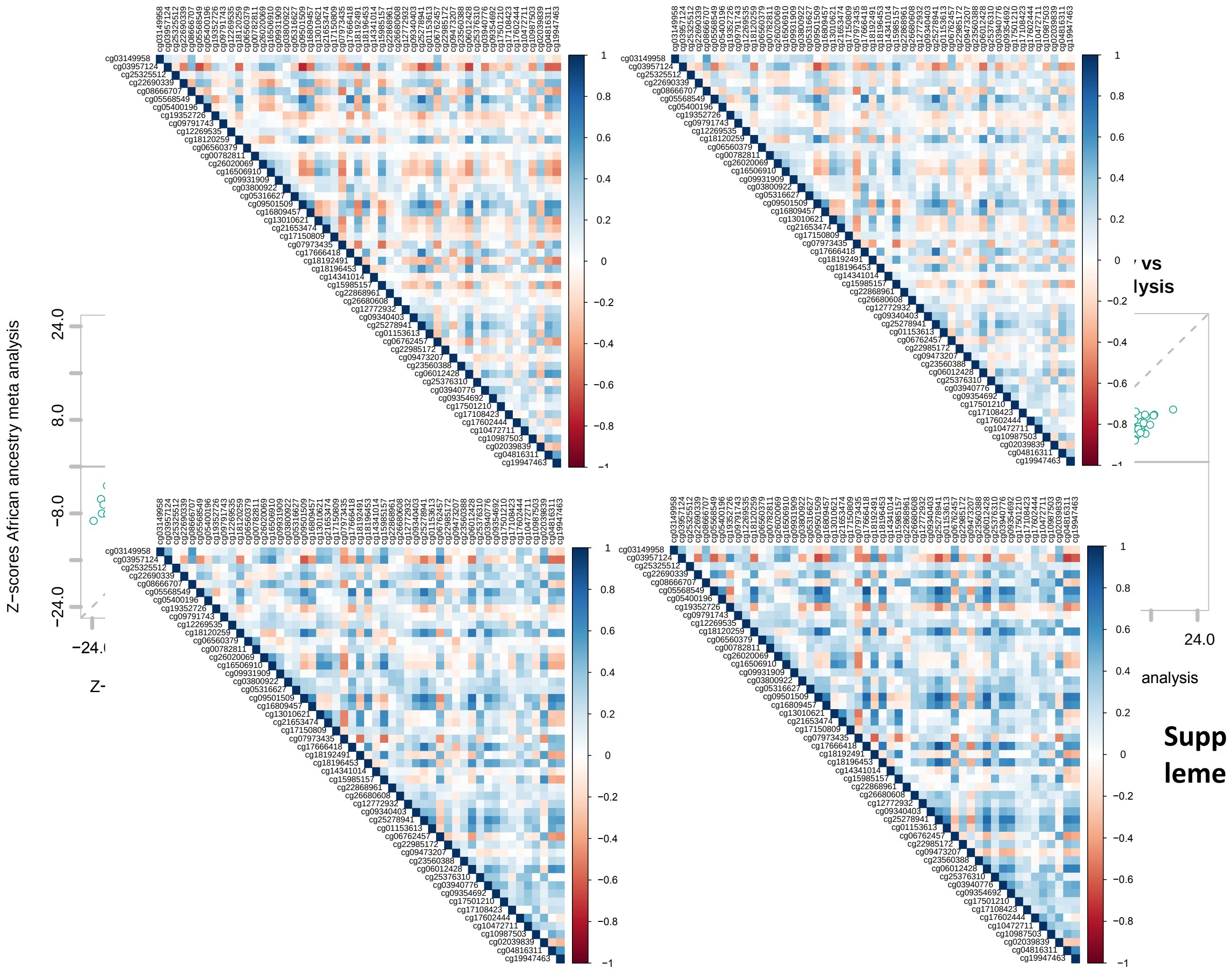

ntary Figure 7: Transethnic meta-analysis. For the 1,765 Bonferroni significant marker we compared Z-scores of each ancestry to transethnic discovery meta-analysis. Z-scores were calculated dividing effect sizes through standard errors.

Supplementary Figure 8: Correlation of coefficients for DNA methylation values. From top left to bottom right cohorts are AIRWAVE, KORA, NFBC1966 and NFBC1986. Pearson correlation coefficients of random genomic region including 50 CRP-associated CpGs (see method section Correlation within CRP-associated markers). The 50 CpGs are the same in all

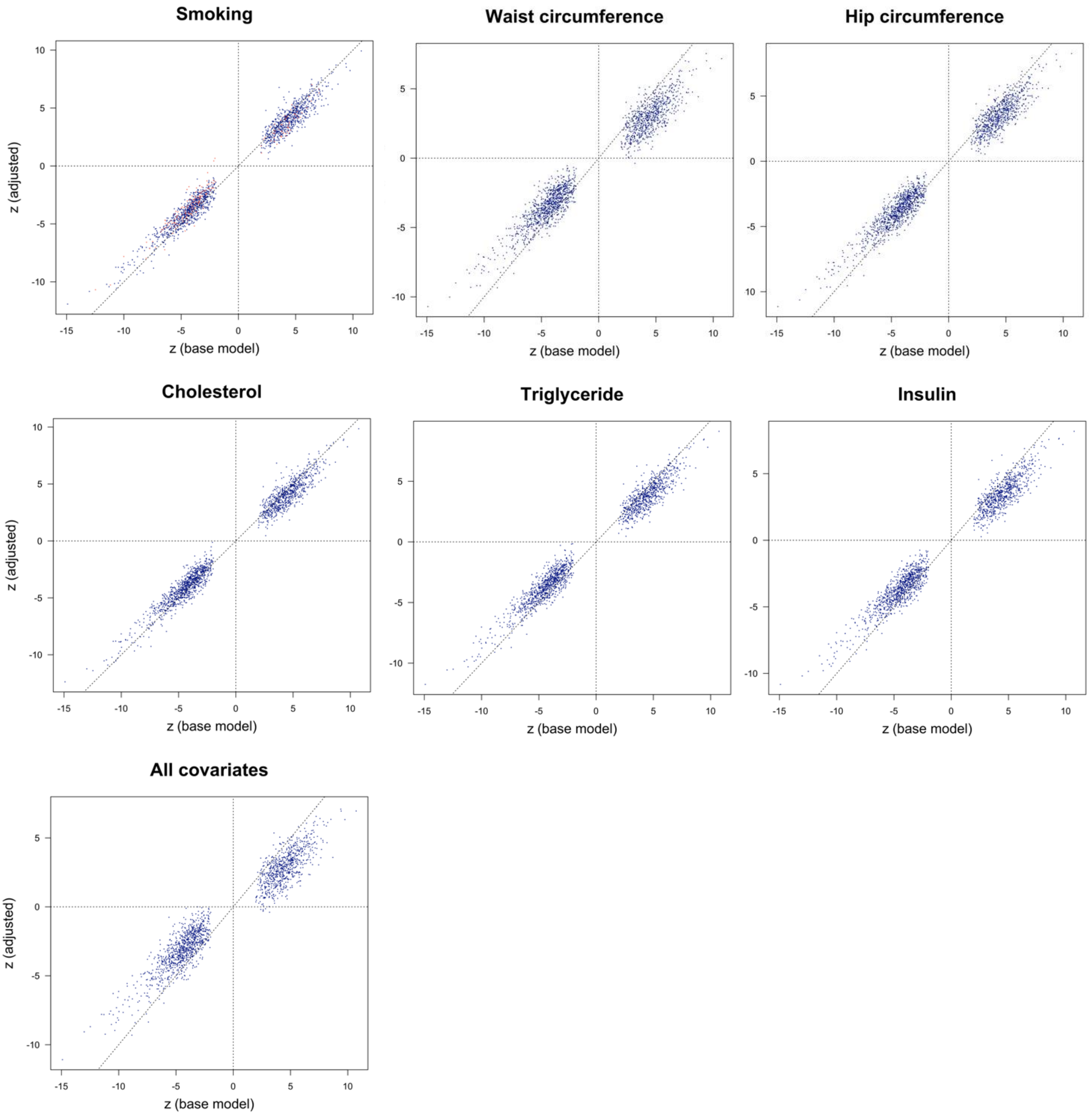

4 datasets. All CpGs are sorted according their chromosomal position. Airwave data were generated on EPIC arrays whereas all other data was derived from Illumina 450k arrays.

Supplementary Figure 9: Z-score comparison from sensitivity analysis. From top left to bottom: smoking, waist circumference, hip circumference, total cholesterol, triglycerides, insulin, all tested covariates in one per regression model. For example the two models compared in the top left plot were: log(CRP) ~ DNAmeth + age + sex + estimated blood cell count + technical covariates as the base model plotted on the x axis versus Z scores from the model log(CRP) ~ DNAmeth + age + sex + estimated blood cell count + technical covariates + smoking on the y axis.

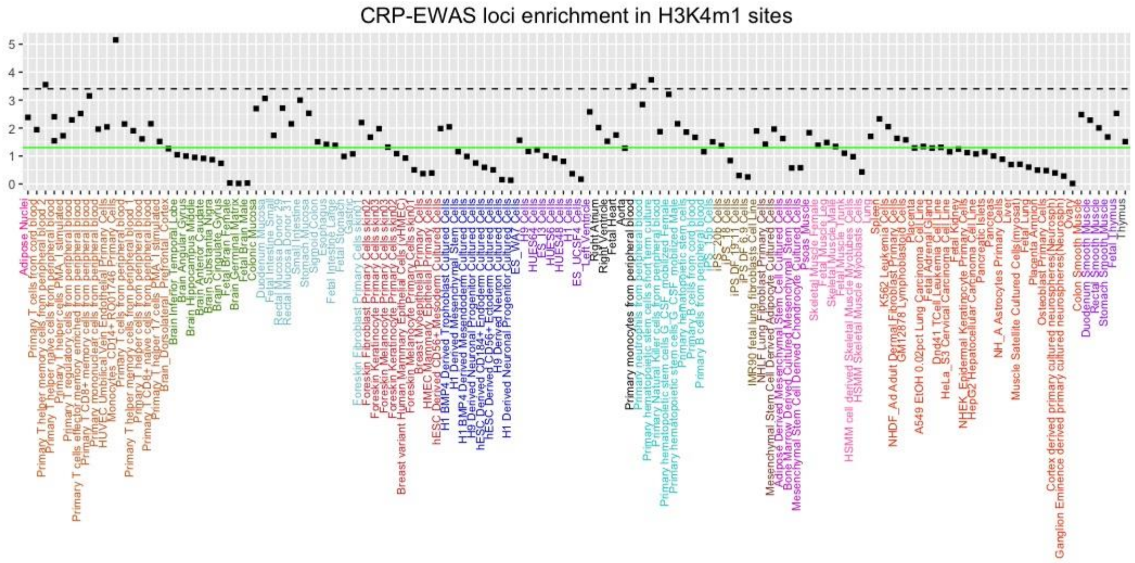

Supplementary Figure 10: Result of overrepresentation analysis for H3K4me1 across roadmap tissues. On the x axis datasets are given. Each entry represents one H3K4me1 data set for one specific tissue. The tissues are grouped by color. Y axis gives the negative log10 value of Empirical P-values for the overlap derived from a permutation test (described in more detail in method section “Overrepresentation analysis”).

Green line is P = 0.05; dotted line is Bonferroni significance level for all tested tissues. P value of overlap is derived from complete 1511 loci list.

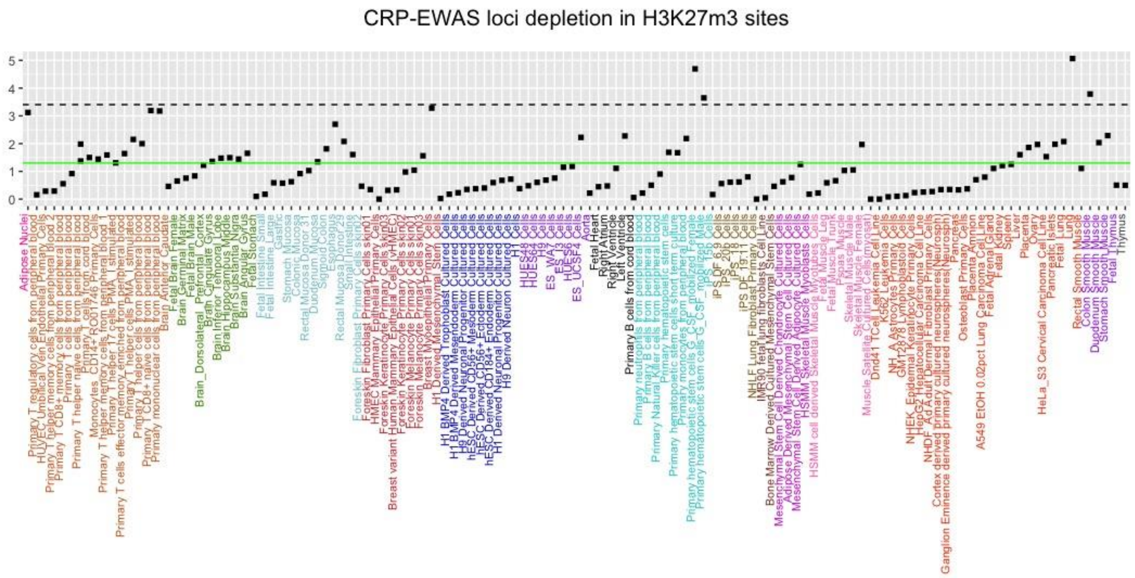

Supplementary Figure 11: Result of overrepresentation analysis for H3K27me3 across roadmap tissues. On the x axis datasets are given. Each entry represents one H3K27me3 data set for one specific tissue. The tissues are grouped by color. Y axis gives the negative log10 value of Empirical P-values for the overlap derived from a permutation test (described in more detail in method section “Overrepresentation analysis”).

Green line is P = 0.05; dotted line is the Bonferroni significance level for all tested tissues. P value of overlap is derived from the complete 1511 loci list.

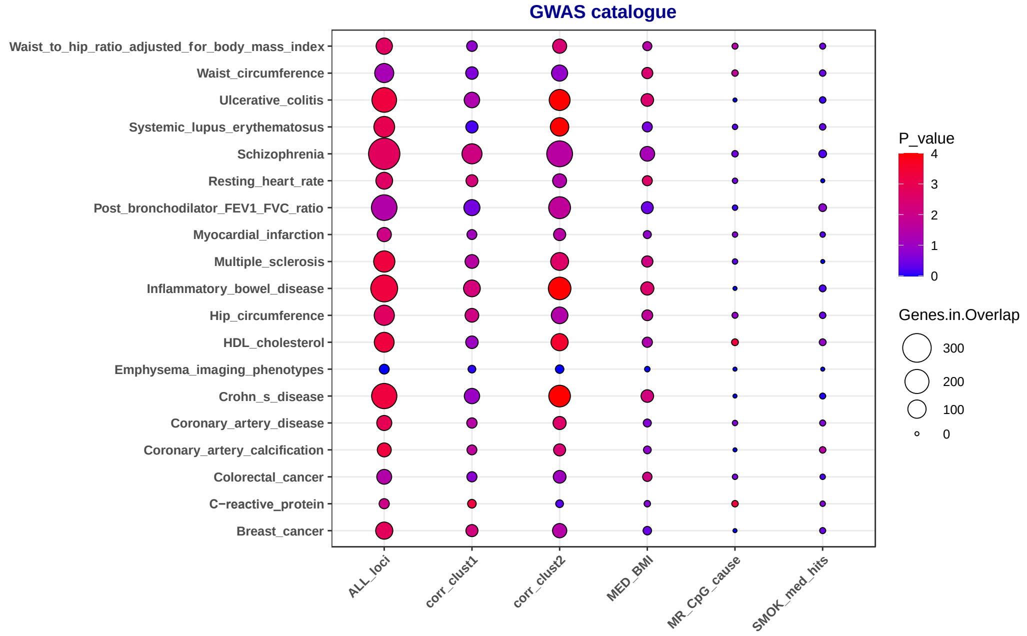

Supplementary Figure 9: Result of overrepresentation analysis. Selected traits from overlap between GWAS catalogue and CRP associated CpG signatures. Detailed explanation of overlap calculation and included trait is given in online methods.

BIOS Consortium

Management Team
Bastiaan T. Heijmans (chair)[76], Peter A.C. ’t Hoen[87], Joyce van Meurs[3], Rick Jansen[89], Lude Franke[90].

Cohort collection
Dorret I. Boomsma[91], René Pool[91], Jenny van Dongen[91], Jouke J. Hottenga[91] (Netherlands Twin Register); Marleen MJ van Greevenbroek[92], Coen D.A. Stehouwer[92], Carla J.H. van der Kallen[92], Casper G. Schalkwijk[92] (Cohort study on Diabetes and Atherosclerosis Maastricht); Cisca Wijmenga[90], Lude Franke[90], Sasha Zhernakova[90], Ettje F. Tigchelaar[90] (LifeLines Deep); P. Eline Slagboom[76], Marian Beekman[76], Joris Deelen[76], Diana van Heemst[93] (Leiden Longevity Study); Jan H. Veldink[94], Leonard H. van den Berg[94] (Prospective ALS Study Netherlands); Cornelia M. van Duijn[90], Bert A. Hofman[22], Aaron Isaacs[90], André G. Uitterlinden[3] (Rotterdam Study).

Data Generation
Joyce van Meurs (Chair)[3], P. Mila Jhamai[3], Michael Verbiest[3], H. Eka D. Suchiman[76], Marijn Verkerk[3], Ruud van der Breggen[76], Jeroen van Rooij, Nico Lakenberg[76].

Data management and computational infrastructure
Hailiang Mei (Chair)[95], Maarten van Iterson[76], Michiel van Galen[87], Jan Bot[95], Dasha V. Zhernakova[90], Rick Jansen[89], Peter van ’t Hof[95], Patrick Deelen[90], Irene Nooren[95], Peter A.C. ’t Hoen[87], Bastiaan T. Heijmans[76], Matthijs Moed[76].

Data Analysis Group
Lude Franke (Co-Chair)[90], Martijn Vermaat[2], Dasha V. Zhernakova[90], René Luijk[76], Marc Jan Bonder[90], Maarten van Iterson[76], Patrick Deelen[90], Freerk van Dijk[97], Michiel van Galen[88], Wibowo Arindrarto[95], Szymon M. Kielbasa[98], Morris A. Swertz[97], Erik. W van Zwet[98], Rick Jansen[89], Peter-Bram ’t Hoen (Co-Chair)[88], Bastiaan T. Heijmans (Co-Chair)[76].

3 Department of Internal Medicine, ErasmusMC, Rotterdam, The Netherlands

22 Department of Epidemiology, ErasmusMC, Rotterdam, The Netherlands

76 Molecular Epidemiology, Department of Biomedical Data Sciences, Leiden University Medical Center, Leiden, The Netherlands

87 Department of Human Genetics, Leiden University Medical Center, Leiden, The Netherlands

88 Department of Genetic Epidemiology, ErasmusMC, Rotterdam, The Netherlands

89 Department of Psychiatry, VU University Medical Center, Neuroscience Campus Amsterdam, Amsterdam, The Netherlands

90 Department of Genetics, University of Groningen, University Medical Centre Groningen, Groningen, The Netherlands

91 Department of Biological Psychology, VU University Amsterdam, Neuroscience Campus Amsterdam, Amsterdam, The Netherlands

92 Department of Internal Medicine and School for Cardiovascular Diseases (CARIM), Maastricht University Medical Center, Maastricht, The Netherlands

93 Department of Gerontology and Geriatrics, Leiden University Medical Center, Leiden, The Netherlands

94 Department of Neurology, Brain Center Rudolf Magnus, University Medical Center Utrecht, Utrecht, The Netherlands

95 Sequence Analysis Support Core, Department of Biomedical Data Sciences, Leiden University Medical Center, Leiden, The Netherlands

96 SURFsara, Amsterdam, the Netherlands

97 Genomics Coordination Center, University Medical Center Groningen, University of Groningen, Groningen, the Netherlands

98 Medical Statistics, Department of Biomedical Data Sciences, Leiden University Medical Center, Leiden, The Netherlands

References

1. Elliott, P. *et al.* The Airwave Health Monitoring Study of police officers and staff in Great Britain: rationale, design and methods. *Environ Res* **134**, 280-5 (2014).
2. Fiorito, G. *et al.* Social adversity and epigenetic aging: a multi-cohort study on socioeconomic differences in peripheral blood DNA methylation. *Sci Rep* **7**, 16266 (2017).
3. Chen, Y.A. *et al.* Discovery of cross-reactive probes and polymorphic CpGs in the Illumina Infinium HumanMethylation450 microarray. *Epigenetics* **8**, 203-9 (2013).
4. Boyd, A. *et al.* Cohort Profile: the 'children of the 90s'--the index offspring of the Avon Longitudinal Study of Parents and Children. *Int J Epidemiol* **42**, 111-27 (2013).
5. Golding, J., Pembrey, M., Jones, R. & Team, A.S. ALSPAC--the Avon Longitudinal Study of Parents and Children. I. Study methodology. *Paediatr Perinat Epidemiol* **15**, 74-87 (2001).
6. Fraser, A. *et al.* Cohort Profile: the Avon Longitudinal Study of Parents and Children: ALSPAC mothers cohort. *Int J Epidemiol* **42**, 97-110 (2013).
7. The Atherosclerosis Risk in Communities (ARIC) Study: design and objectives. The ARIC investigators. *Am J Epidemiol* **129**, 687-702 (1989).
8. Chu, A.Y. *et al.* Epigenome-wide association studies identify DNA methylation associated with kidney function. *Nat Commun* **8**, 1286 (2017).
9. Teschendorff, A.E. *et al.* A beta-mixture quantile normalization method for correcting probe design bias in Illumina Infinium 450 k DNA methylation data. *Bioinformatics* **29**, 189-96 (2013).
10. Ikram, M.A. *et al.* Objectives, design and main findings until 2020 from the Rotterdam Study. *Eur J Epidemiol* **35**, 483-517 (2020).
11. Schram, M.T. *et al.* The Maastricht Study: an extensive phenotyping study on determinants of type 2 diabetes, its complications and its comorbidities. *Eur J Epidemiol* **29**, 439-51 (2014).
12. Schoenmaker, M. *et al.* Evidence of genetic enrichment for exceptional survival using a family approach: the Leiden Longevity Study. *Eur J Hum Genet* **14**, 79-84 (2006).
13. Tigchelaar, E.F. *et al.* Cohort profile: LifeLines DEEP, a prospective, general population cohort study in the northern Netherlands: study design and baseline characteristics. *BMJ Open* **5**, e006772 (2015).
14. Scholtens, S. *et al.* Cohort Profile: LifeLines, a three-generation cohort study and biobank. *Int J Epidemiol* **44**, 1172-80 (2015).
15. Willemsen, G. *et al.* The Adult Netherlands Twin Register: twenty-five years of survey and biological data collection. *Twin Res Hum Genet* **16**, 271-81 (2013).
16. van Greevenbroek, M.M. *et al.* Human plasma complement C3 is independently associated with coronary heart disease, but only in heavy smokers (the CODAM study). *Int J Cardiol* **154**, 158-62 (2012).
17. Huisman, M.H. *et al.* Population based epidemiology of amyotrophic lateral sclerosis using capture-recapture methodology. *J Neurol Neurosurg Psychiatry* **82**, 1165-70 (2011).

18. Deelen, J. *et al.* Genome-wide association meta-analysis of human longevity identifies a novel locus conferring survival beyond 90 years of age. *Hum Mol Genet* **23**, 4420-32 (2014).
19. Simons, N. *et al.* A Common Gene Variant in Glucokinase Regulatory Protein Interacts With Glucose Metabolism on Diabetic Dyslipidemia: the Combined CODAM and Hoorn Studies. *Diabetes Care* **39**, 1811-7 (2016).
20. van Rheenen, W. *et al.* Genome-wide association analyses identify new risk variants and the genetic architecture of amyotrophic lateral sclerosis. *Nat Genet* **48**, 1043-8 (2016).
21. Deelen, P. *et al.* Genotype harmonizer: automatic strand alignment and format conversion for genotype data integration. *BMC Res Notes* **7**, 901 (2014).
22. Whole-genome sequence variation, population structure and demographic history of the Dutch population. *Nat Genet* **46**, 818-25 (2014).
23. Howie, B.N., Donnelly, P. & Marchini, J. A flexible and accurate genotype imputation method for the next generation of genome-wide association studies. *PLoS Genet* **5**, e1000529 (2009).
24. Fried, L.P. *et al.* The Cardiovascular Health Study: design and rationale. *Ann Epidemiol* **1**, 263-76 (1991).
25. Aryee, M.J. *et al.* Minfi: a flexible and comprehensive Bioconductor package for the analysis of Infinium DNA methylation microarrays. *Bioinformatics* **30**, 1363-9 (2014).
26. Smith, A.K. *et al.* Epigenetic changes associated with inflammation in breast cancer patients treated with chemotherapy. *Brain Behav Immun* **38**, 227-36 (2014).
27. Barfield, R.T., Kilaru, V., Smith, A.K. & Conneely, K.N. CpGassoc: an R function for analysis of DNA methylation microarray data. *Bioinformatics* **28**, 1280-1 (2012).
28. Bendinelli, B. *et al.* Fruit, vegetables, and olive oil and risk of coronary heart disease in Italian women: the EPICOR Study. *Am J Clin Nutr* **93**, 275-83 (2011).
29. Palli, D. *et al.* A molecular epidemiology project on diet and cancer: the EPIC-Italy Prospective Study. Design and baseline characteristics of participants. *Tumori* **89**, 586-93 (2003).
30. Zhang, Y. *et al.* DNA methylation signatures in peripheral blood strongly predict all-cause mortality. *Nat Commun* **8**, 14617 (2017).
31. Leitsalu, L. *et al.* Cohort Profile: Estonian Biobank of the Estonian Genome Center, University of Tartu. *Int J Epidemiol* **44**, 1137-47 (2015).
32. Lehne, B. *et al.* A coherent approach for analysis of the Illumina HumanMethylation450 BeadChip improves data quality and performance in epigenome-wide association studies. *Genome Biol* **16**, 37 (2015).
33. Holle, R., Happich, M., Lowel, H., Wichmann, H.E. & Group, M.K.S. KORA--a research platform for population based health research. *Gesundheitswesen* **67 Suppl 1**, S19-25 (2005).
34. Wichmann, H.E., Gieger, C., Illig, T. & Group, M.K.S. KORA-gen--resource for population genetics, controls and a broad spectrum of disease phenotypes. *Gesundheitswesen* **67 Suppl 1**, S26-30 (2005).
35. Zeilinger, S. *et al.* Tobacco smoking leads to extensive genome-wide changes in DNA methylation. *PLoS One* **8**, e63812 (2013).
36. Houseman, E.A. *et al.* DNA methylation arrays as surrogate measures of cell mixture distribution. *BMC Bioinformatics* **13**, 86 (2012).

37. Deary, I.J., Gow, A.J., Pattie, A. & Starr, J.M. Cohort profile: the Lothian Birth Cohorts of 1921 and 1936. *Int J Epidemiol* **41**, 1576-84 (2012).
38. Deary, I.J. *et al.* The Lothian Birth Cohort 1936: a study to examine influences on cognitive ageing from age 11 to age 70 and beyond. *BMC Geriatr* **7**, 28 (2007).
39. Deary, I.J., Whiteman, M.C., Starr, J.M., Whalley, L.J. & Fox, H.C. The impact of childhood intelligence on later life: following up the Scottish mental surveys of 1932 and 1947. *J Pers Soc Psychol* **86**, 130-47 (2004).
40. Taylor, A.M., Pattie, A. & Deary, I.J. Cohort Profile Update: The Lothian Birth Cohorts of 1921 and 1936. *Int J Epidemiol* **47**, 1042-1042r (2018).
41. Shah, S. *et al.* Genetic and environmental exposures constrain epigenetic drift over the human life course. *Genome Res* **24**, 1725-33 (2014).
42. McIlhagger, R. *et al.* Differences in the haematological profile of healthy 70 year old men and women: normal ranges with confirmatory factor analysis. *BMC Blood Disord* **10**, 4 (2010).
43. Rantakallio, P. The longitudinal study of the northern Finland birth cohort of 1966. *Paediatr Perinat Epidemiol* **2**, 59-88 (1988).
44. Sovio, U. *et al.* Genetic determinants of height growth assessed longitudinally from infancy to adulthood in the northern Finland birth cohort 1966. *PLoS Genet* **5**, e1000409 (2009).
45. Ikram, M.A. *et al.* The Rotterdam Study: 2018 update on objectives, design and main results. *Eur J Epidemiol* **32**, 807-850 (2017).
46. Volzke, H. *et al.* Cohort profile: the study of health in Pomerania. *Int J Epidemiol* **40**, 294-307 (2011).
47. Verdi, S. *et al.* TwinsUK: The UK Adult Twin Registry Update. *Twin Res Hum Genet* **22**, 523-529 (2019).
48. Tsaprouni, L.G. *et al.* Cigarette smoking reduces DNA methylation levels at multiple genomic loci but the effect is partially reversible upon cessation. *Epigenetics* **9**, 1382-96 (2014).
49. Triche, T.J., Jr., Weisenberger, D.J., Van Den Berg, D., Laird, P.W. & Siegmund, K.D. Low-level processing of Illumina Infinium DNA Methylation BeadArrays. *Nucleic Acids Res* **41**, e90 (2013).
