# Supplementary material for: DNA methylation signature of chronic low-grade inflammation and its role in cardio-respiratory diseases
Source: Nat Commun. 2022 May 3;13:2408. doi: 10.1038/s41467-022-29792-6 (PMC9065016; doi:10.1038/s41467-022-29792-6)
Supplement: Supplementary file 3 — Description of Additional Supplementary Files [file 41467_2022_29792_MOESM3_ESM.pdf]

File Name: Supplementary Data 1:
Description: QC, data cleaning on individual cohorts
File Name: Supplementary Data 2:
Description: marker list result overview
File Name: Supplementary Data 3:
Description: results of bias corrected meta analysis
File Name: Supplementary Data 4:
Description: ancestry specific marker
File Name: Supplementary Data 5:
Description: sensitivity analysis
File Name: Supplementary Data 6:
Description: Mendelian randomisation: CpG causes CRP changes - IVW results
File Name: Supplementary Data 7:
Description: Mendelian randomisation: CpG causes CRP changes - Triangulation
File Name: Supplementary Data 8:
Description: Mendelian randomisation: CRP causes CpG changes - IVW results
File Name: Supplementary Data 9:
Description: Mendelian randomisation: CRP causes CpG changes - Triangulation
File Name: Supplementary Data 10:
Description: Mediation results in NFBC1966 (6 models)
File Name: Supplementary Data 11:
Description: Mediation results: model BMI via CRP to CpG
File Name: Supplementary Data 12:
Description: Mediation results: model SMOKING via CRP to CpG
File Name: Supplementary Data 13:
Description: over representation analysis: overlap to chromosomal position
File Name: Supplementary Data 14:
Description: over representation analysis: Roadmap Chromatin model
File Name: Supplementary Data 15:
Description: over representation analysis: Roadmap Histone marks: H3K4
File Name: Supplementary Data 16:
Description: over representation analysis: Roadmap Histone marks: H3K27
File Name: Supplementary Data 17:
Description: over representation analysis: HiC
File Name: Supplementary Data 18:
Description: over representation analysis: Encode transcription factors
File Name: Supplementary Data 19:
Description: GxE significant assocations
File Name: Supplementary Data 20:
Description: over representation analysis: overlap to GWAS catalogue
File Name: Supplementary Data 21:
Description: over representation analysis: overlap to EWAS catalogue 1 (BIG)
File Name: Supplementary Data 22:
Description: over representation analysis: overlap to EWAS catalogue 2 (MRC)
File Name: Supplementary Data 23:
Description: CRP risk score analysis.
